# Reducing Ensembles of Protein Tertiary Structures Generated De Novo via Clustering

**DOI:** 10.3390/molecules25092228

**Published:** 2020-05-09

**Authors:** Ahmed Bin Zaman, Parastoo Kamranfar, Carlotta Domeniconi, Amarda Shehu

**Affiliations:** 1 Department of Computer Science, George Mason University, Fairfax, VA 22030, USA; azaman6@gmu.edu (A.B.Z.); pkamranf@gmu.edu (P.K.); 2 Center for Advancing Human-Machine Partnerships, George Mason University, Fairfax, VA 22030, USA; 3 Department of Bioengineering, George Mason University, Fairfax, VA 22030, USA; 4 School of Systems Biology, George Mason University, Fairfax, VA 22030, USA

**Keywords:** protein structure prediction, tertiary structure, decoy ensemble, reduction, clustering

## Abstract

Controlling the quality of tertiary structures computed for a protein molecule remains a central challenge in de-novo protein structure prediction. The rule of thumb is to generate as many structures as can be afforded, effectively acknowledging that having more structures increases the likelihood that some will reside near the sought biologically-active structure. A major drawback with this approach is that computing a large number of structures imposes time and space costs. In this paper, we propose a novel clustering-based approach which we demonstrate to significantly reduce an ensemble of generated structures without sacrificing quality. Evaluations are related on both benchmark and CASP target proteins. Structure ensembles subjected to the proposed approach and the source code of the proposed approach are publicly-available at the links provided in Section 1.

## 1. Introduction

The three-dimensional/tertiary structure of a protein molecule is key to determining its array of activities in the cell, as proteins employ their tertiary structures to interface with other molecular partners [1]. Though the path to decoding protein function seems to go through its tertiary structure, determining the biologically-active structure(s) of a protein poses many challenges in both the wet and dry laboratory [2]. Great progress has been made in wet-laboratory structure determination, but these advancements nonetheless lag behind the technological advances in gene sequencing; the increasingly faster and cheaper high-throughput gene sequencing technologies have yielded millions of protein sequences [3]. In contrast, the number of known biologically-active/native protein structures is an order of magnitude less. For instance, as of April 2020, the number of experimentally-known structures deposited in the Protein Data Bank (PDB) [4] is around 160,000.

The above discrepancy continues to motivate protein structure prediction (PSP) in dry laboratories. One of the most challenging settings is that of de-novo/template-free PSP, where the only direct information about a target protein at hand is its amino-acid sequence [5]. This is typically the case for proteins that do not have other, sufficiently-similar protein sequences with known structures that could otherwise serve as structural templates upon which to thread the target sequence [5]. It is worth noting that there is some evidence of stagnation in the rate of discovery of new protein folds [6]. This suggests that reasonable structural templates may soon be found for increasingly many more target proteins and transferred onto them via homology modeling.

Prediction in a de-novo setting is carried out in two stages. In the first stage, the focus is on computing or generating physically-realistic structures. Structure generation algorithms address an optimization problem; they seek tertiary structures that minimize the interaction energy among the atoms of a given target protein. It is now well-known that the energy functions designed in computational laboratories are inherently inaccurate [7,8,9]. In particular, one cannot infer that a lower-energy structure is more similar to the sought native structure than a higher-energy structure. In a blind setting, it is unknown which structures are sufficiently close to the sought native structure and can be deemed near-native. The objective of the second stage is to determine such structures. The algorithms put forth for this purpose are known as model assessment, model selection, or decoy selection; the terms “model” and “decoy” refer to a computed/generated structure in this context.

Great algorithmic advances have been made in structure generation, most remarkably by Rosetta [10], Quark [11], and others [12,13,14,15,16]. Recent works have investigated incorporating complementary information like sequence-predicted contacts and constructing new energy functions based on predicted contacts or distances of pairs of amino acids for structure generation [17,18,19]. Algorithms utilizing deep neural networks are also becoming increasingly popular [20,21,22,23,24]. Such advances are documented in the Critical Assessment of protein Structure Prediction (CASP), which is a biennial community experiment/competition that assesses progress in PSP in several categories, including the template-free category (also referred to as free modeling) [25].

Outstanding challenges concern how to control the quality of generated structures. Despite clever choices in representing tertiary structures [5,26], the search space that has to be explored by a structure generation algorithm is vast and high-dimensional. Consider the following back-of-the-envelope calculation: on a protein not exceeding 100 amino acids, modeling three backbone dihedral angles per amino acids results in around 300 variables; these constitute the 300 dimensions of the search space. In addition, the energy function that structure generation algorithms optimize (by seeking structures that reside in local minima of the energy surface) is noisy.

Currently, when employing popular platforms, such as Rosetta and Quark, the recommendation from developers is to generate as many structures that can be afforded, with actual numbers practically varying between 10,000 and 20,000 structures. This recommendation acknowledges that more structures may translate into higher likelihood that some will reside near the sought native structure. The recommendation is impractical for various reasons. While generating structures used to be significantly more expensive than analyzing them, now this relationship is less imbalanced. Great progress in software and hardware have made it less costly to generate structures. For instance, algorithms operating under the umbrella of evolutionary computation can generate hundreds of thousands of structures [12,13,16,17]. Algorithms tasked with analyzing these structures now may have to additionally deal with a data size issue.

Moreover, in most template-free PSP protocols, the end of the first stage adds back the side-chain atoms on each structure and carries out local improvements on the resulting all-atom structures prior to handing them off to the second stage. Adding atomistic detail on a structure is computationally expensive, as the energy function employed has to handle a large number of atoms per structure (that includes all side-chain atoms and all hydrogen atoms for each amino acid). This is exacerbated when the recommendation is to collect large numbers of structures.

In this paper, we propose a novel approach to reduce the size of a structure ensemble. The objective is to do so while retaining the quality of the original ensemble, as measured via several metrics described later in the manuscript in Section 2. To the best of our knowledge, the problem of structure ensemble reduction while preserving quality is unexplored. The problem is also not trivial. In the de-novo setting, it may be tempting to tackle it by discarding higher-energy structures. Indeed, early work in [27] does so before proceeding to cluster the remaining structures for the purpose of model selection. As we show in our evaluation in Section 3, an approach that simply utilizes an energy threshold, which we employ as a baseline for the purpose of comparison, does a poor job at retaining near-native structures. This is not surprising. As the above discussion relates, energy is not a reliable indicator of nativeness.

The proposed approach seeks to retain the inherent organization of the structures in a given ensemble and does so by utilizing clustering algorithms. While this paper evaluates the impact of various clustering algorithms, the contribution is not in proposing clustering algorithms; nor is the focus of this paper in evaluating the vast landscape of existing clustering algorithms. Instead, the contribution of the proposed approach is in leveraging smart featurization of tertiary structures, computationally-expedient clustering in the feature space to capture the inherent organization, and selecting a subset of structures that retain the exposed organization in tandem with additional properties of interest. The latter here are limited to internal energies, but other properties of interest may be utilized that may expand the applicability of the proposed approach to structure ensembles beyond the de-novo setting. The main reason we focus on such ensembles here is due to our ability to generate many structures and the clear need for the proposed approach in de-novo PSP.

It is also worth noting that the efforts described in this manuscript are not targeting the problem of model assessment/selection, though the reduced ensembles may be of use downstream to methods that tackle model assessment/selection. Some of these methods employ clustering, supervised learning, or a combination of learning techniques [27,28,29,30,31,32,33]. A preliminary version of this work has appeared in [34]. Here we expand the work by adding clustering algorithms, metrics measuring quality, and detailed evaluations. Rigorous analysis is conducted to determine the optimal settings for the employed clustering algorithms. Evaluations are carried out on diverse target protein datasets that include recent hard, free-modeling domains from CASP12 and CASP13 and show that the proposed approach yields drastic reductions in ensemble size while retaining structure quality.

We note that the proposed approach is publicly-available at https://github.com/psp-codes/reduced-decoy-ensemble (Code doi:10.5281/zenodo.3758031). All structure ensembles subjected to the proposed approach are provided freely on IEEEDataPort at https://ieee-dataport.org/open-access/protein-tertiary-structures-zamanmolecules20 (Data doi:10.21227/gq2v-8k24).

The rest of this paper is organized as follows. We describe the proposed methodology in Section 2. Evaluation is presented in Section 3. The paper concludes with a summary and discussion of future work in Section 5.

## 2. Materials and Methods

We will refer to a given ensemble of structures as $\Omega_{\mathrm{gen}}$ and to the reduced ensemble (by our approach) as $\Omega_{\mathrm{red}}$. We note that $\Omega_{\mathrm{red}}$ keeps only a fraction of the structures in the original $\Omega_{\mathrm{gen}}$. To efficiently produce a reduced-size structure ensemble $\Omega_{\mathrm{red}}$ that retains the quality and diversity of the original, full-size structure ensemble $\Omega_{\mathrm{gen}}$, the method we propose in this paper leverages fast shape similarity for tertiary structures.

The proposed method consists of three stages. First, a *featurizer* extracts structure features that summarize the three-dimensional shape. Second, the features are then utilized by a clustering algorithm to group structures based on their shape similarity. Third and finally, a *selector* selects structures from each cluster/group identified over $\Omega_{\mathrm{gen}}$ to populate the reduced ensemble $\Omega_{\mathrm{red}}$. In the following, we describe each of these stages in detail. Before doing so, in the interest of clarity, we provide some more details into how the structures we utilize to evaluate our method are generated in the first place.

### 2.1. Generation of Structures for a Target Protein

Many options are available to generate the ensemble $\Omega_{\mathrm{gen}}$, Rosetta, Quark, etc. We choose to utilize an in-house algorithm, the hybrid evolutionary algorithm (HEA) [16], which has been evaluated against Rosetta and other algorithms [14,15,16]. HEA leverages evolutionary search techniques to balance between exploration and exploitation in a vast search space and has been shown to have higher exploration capability than Rosetta [15,16]. While any structure generation algorithm can be used to generate the $\Omega_{\mathrm{gen}}$ ensemble for our purposes in this paper, we specifically employ HEA due to its high exploration capability; the algorithm can generate hundreds of thousands of structures for a target protein (given its amino-acid sequence) in a matter of hours.

In its functional core, the HEA builds over the Rosetta engine and utilizes its representation of structures, as well its suite of energy functions. In summary, HEA evolves a fixed-size population of structures over a number of generations. The initial population is constructed by first creating identical extended chains from the amino-acid sequence of a target protein and then randomizing the chains by employing repeated molecular fragment replacements of length 9. The molecular fragment replacement operation in HEA utilizes libraries of fragments of length 9 and 3 generated for a given amino-acid sequence via the Robetta server [10]. The fragments are excised from known native structures of proteins in the PDB. To perform molecular fragment replacement on a structure, first an amino acid index *i* is chosen at random from the range $\left[1,L_{p}-L_{f}+1\right]$, where $L_{p}$ is the number of amino acids in a given sequence, and $L_{f}$ is the length of fragment. The fragment composed of amino acids $\left[i,i+L_{f}-1\right]$ in the given structure is then replaced with a fragment configuration selected at random from the available fragments in the fragment library with the same or similar amino-acid sequence.

In each generation of HEA, the structures in a population are considered parents, and each parent is subjected to molecular fragment replacement of length 3 to produce an offspring. Each offspring is improved by repeatedly applying molecular fragment replacement of length 3. Next, the improved offspring and the top 25% of the parents compete against one another in terms of energy. The top structures survive to become parents for the next generation. Interested readers can learn more about HEA in Ref. [16].

### 2.2. Stage I: Featurizing Generated Structures

We utilize the Ultrafast Shape Recognition (USR) metrics that were originally introduced in [35] to summarize three-dimensional structures of ligands. USR metrics were used in [35] to expedite searches for similar structures in molecular databases. These metrics have also been used by us and others to expedite robotics-inspired algorithms exploring protein structure spaces for structure [36,37] and motion computation [38,39].

**Figure 1:**
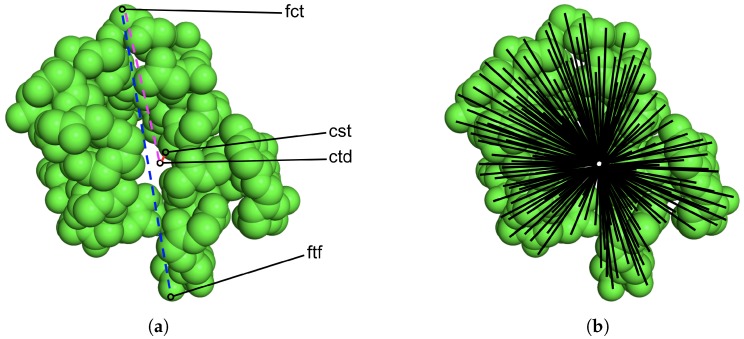
(**a**) The four reference points used to calculate USR metrics on a given tertiary structure are shown here. (**b**) Distances of all atoms from a chosen reference point, the ctd, are also shown.

In this paper, we use the USR metrics as features to summarize a tertiary structure. The metrics allow efficient comparison of molecular shapes. They summarize the distributions of distances of all atoms in a tertiary structure from four chosen reference points: the molecular centroid (ctd), the closest atom to ctd (cst), the farthest atom to ctd (fct), and the farthest atom to fct (ftf). Figure 1a shows the locations of these four reference points, with atoms drawn as spheres in a tertiary structure selected for illustration. Once the reference points have been calculated, distances of all atoms from each reference point are calculated next; Figure 1b shows distances of all atoms from one of the reference points, the ctd, by drawing them as lines. The moments of the calculated distance distributions are recorded to summarize a given tertiary structure. Specifically, in our work (as originally in [35]), the resulting distributions are summarized with three momenta, the mean, variance, and skewness. Hence, each tertiary structure in $\Omega_{\mathrm{gen}}$ is represented by 12 features.

The motivation of encoding each tertiary structure via features is three-fold. First, a lower number of coordinates required to represent each structure reduces the computational time of any algorithm expected to process the generated structures. Second, high data dimensionality has a negative impact on the performance of clustering algorithms [40,41,42]. Third, unlike representations based on Cartesian coordinates, the USR-based representation is invariant to rigid-body motions (translation and rotation in 3D space).

### 2.3. Stage II: Clustering Featurized Structures

The featurized structures are subjected to a clustering algorithm. We evaluate four clustering algorithms, three popular, representative clustering algorithms, k-means, Gaussian Mixture Model (GMM), and hierarchical clustering, and a variant of the gmx-cluster algorithm in the GROningen MAchine for Chemical Simulations (GROMACS) package [43]. The latter has been shown to be effective in clustering protein structures [44].

We briefly summarize each algorithm next, paying more attention to describing how we optimize their parameters and apply them to the featurized structures.

In k-means, the number of clusters *k* is a hyper-parameter. The structures that can serve as cluster centroids is another hyper-parameter. We optimize both as follows. For a given value of *k*, *k* structures are initially selected uniformly at random over $\Omega_{\mathrm{gen}}$ to act as the cluster centroids. This induces a particular grouping *C* of the structures, with each structure assigned to the cluster represented by the structure to which it is closest. To evaluate this particular grouping *C*, we calculate the within-cluster scatter loss function: $L\left(C\right)=\frac{1}{2}\sum_{l=1}^{k}\sum_{i\in C_{l}}\sum_{j\in C_{l},j\neq i}D\left(x_{i},x_{j}\right)$, where $D(x_{i},x_{j})$ measures the Euclidean distance between two points/structures $x_{i}\neq x_{j}$ in the same cluster $C_{l}$, where l∈{1,…,k}. One can now vary the structures selected to serve as cluster centroids over iterations and record the selection resulting in the smallest loss. We do so over 10 iterations for a given *k*, randomly selecting structures as initial centroids in each iteration, recording the optimal selection (and associated grouping) for each iteration.

**Figure 2:**
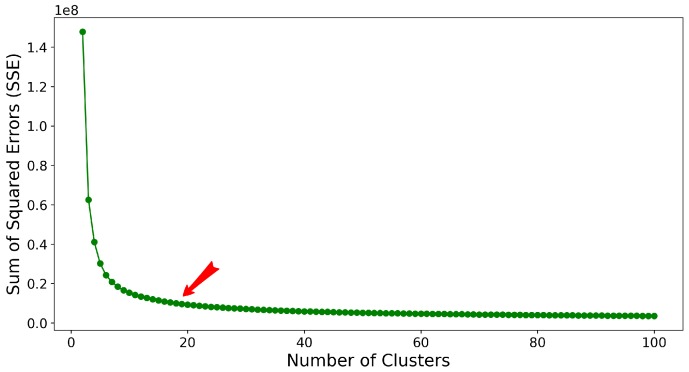
The sum-of-squared errors (SSE) is plotted as a function of the number of clusters *k* identified via k-means on structures generated via HEA on a target protein. This target is part of our evaluation dataset related in Section 3. Specifically, it is the target protein with known native structure in the PDB entry with identifier (id) 1ail. The red arrow points to the knee/elbow region where by increasing *k* SSE does not change noticeably; this is the region from where an optimal value of *k* can be selected.

Note that the above is carried out for a given *k* as *k* varies in a permissive range. To find the optimal number of clusters, *k*, in some considered range, we utilize the popular knee-finding approach [45]. Specifically, after the centroids of clusters are determined (optimally) as above for a given *k*, the squared distance of each structure in a cluster from the centroid of the cluster can be recorded, and the sum of these squared distances can be obtained over the clusters *k* [46]. This sum of squared distances is known as the sum of squared errors (SSE) and is shown for a particular structure dataset in Figure 2. In Figure 2, different values of *k* are plotted against the corresponding SSE values. The knee (also referred to as elbow) in the SSE curve indicates the optimal number of clusters. We are interested in a small value for SSE. Naturally, as one increases *k*, the SSE approaches 0. It is exactly 0 when $k=|\Omega_{\mathrm{gen}}|$ (every structure is the centroid of its own cluster). The goal is to choose a small value of *k* that results in a low SSE. The knee or elbow in the curve that tracks SSE as a function of *k* corresponds to the region where by increasing *k*, SSE does not change noticeably; this is annotated in Figure 2.

GMM is a probabilistic model that assumes a mixture of finite number of Gaussian distributions with unknown parameters as the underling process generation of the data. GMM can be thought of as generalizing k-means, as it includes both information from the covariance structure of the data along with the centers of the Gaussian distributions. The main advantage of GMM is the estimation of uncertainty in data membership to clusters; a conditional probability is assigned to each data indicating the probability with which a specific point belongs to any cluster. As expected, sum of all these conditional probabilities for a given point is 1. This uncertainty assignment makes GMM more informative than k-means [47].

**Figure 3:**
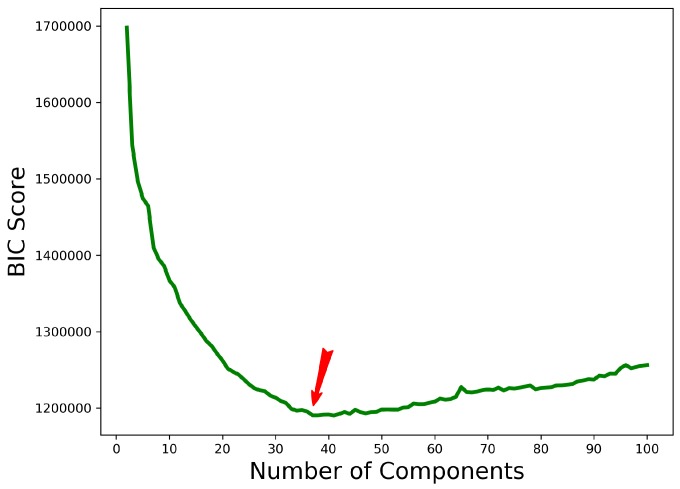
The BIC is plotted as a function of the number of components *k*. Clustering is carried out via GMM on structures generated via HEA on a target protein (known native structure in the PDB entry with identifier (id) 2h5nd). The red arrow points to the value for *k* identified at the lowest BIC value.

However, as in k-means, one needs to specify the number of clusters/components *a priori* in GMM. The optimal value can be determined by minimizing the Bayesian Information Criterion (BIC) [48] metric which considers both covariance type and the number of components. The BIC is a penalty term for the possible likelihood increment when adding more parameters into the model. Specifically, $BIC=\ln\left(n\right)k-2\ln\left(\hat{L}\right)$, where *k* is the number of components, $\hat{L}$ is the maximized value of the likelihood function, and *n* is the number of data points. In Figure 3, we plot the BIC value as the function of the number of components *k* to demonstrate how one can identify a reasonable value for *k* at the lowest BIC value.

Unlike k-means and GMM, hierarchical clustering does not require *a priori* specifying the number of clusters. It refers to a family of clustering algorithms that build a sequence of nested clusters by merging or splitting them successively [49]. We make use of the bottom-up (agglomerative) approach for hierarchical clustering; each structure is first in its own clusters, and then clusters are successively merged until the root of the resulting dendrogram is reached, with a unique cluster containing all the data. The linkage criterion specifies the merge strategy. We select single linkage, where the distance between two clusters is defined as the distance between the two closest points across the two clusters [50].

“Cutting” at different locations of the dendrogram results in different partitions of the dataset into clusters. To avoid recomputation of the clusters, we make use of a cached implementation of hierarchical clustering, where cutting the tree at different places does not impose any further computation. We employ the Davies-Bouldin (DB) index [51] to determine where to cut the dendrogram. DB is as popular clustering validation technique in the absence of ground truth labels. It is computed on features inherent to the dataset and gives a measure of the average similarity of each cluster with its most similar cluster. Specifically, the DB index evaluates the intra-cluster similarity and inter-cluster differences to provide a non-negative score. A lower DB index corresponds to a better separation between the clusters. In our application of hierarchical agglomerative clustering with single linkage, we consider the DB index at every height of the tree, and we select the height that results in the smallest DB as the optimal partition (and optimal corresponding number of clusters) of a structure dataset.

Unlike the above clustering algorithms, the gmx-cluster algorithm determines clusters based on a pre-specified distance cutoff. The algorithm first calculates the pairwise distance between all pairs of structures. For each structure $x_{i}$, the algorithm then counts the number of other structures (neighbors) that are within the distance cutoff. The structure with the highest number of neighbors is then chosen as the central structure and forms a cluster together with all its neighbors. The formed cluster is then removed from the ensemble of structures and the process is repeated for the remaining structures in the ensemble until the ensemble contains no more structures.

The computation of pairwise distances can potentially be very demanding on large datasets, if one were to use the gmx-cluster implementation that uses lRSMD as the distance metric. Our adaptation of this algorithm transfers all neighbor computations in the USR feature space, using Euclidean distance in the USR feature space as a proxy for lRMSD. These distances, to which we refer as USR scores (and analyze in some detail in Section 4), are normalized between 0 and 1, so that we can set a distance cutoff. We set this cutoff to 0.1; our analysis shows that this is a reasonable value. From now on, we will refer to the adaptation of gmx-cluster as gmx-cluster-usr.

### 2.4. Stage III: Selecting Structures to Populate the Reduced Ensemble

After clustering the featurized $\Omega_{\mathrm{gen}}$, the structures are grouped in clusters. The *selector* now selects a subset of structures from each cluster to populate the reduced ensemble $\Omega_{\mathrm{red}}$. The selector makes this decision by considering both the identified clusters and the Rosetta score4 energy function of structures. This function evaluates not only Lennard-Jones interactions, but also short- and long-range hydrogen bonding in a tertiary structure.

The selector we propose organizes the structures in a cluster into levels/bins; the structures placed in the same bin have identical score4 energies up to two digits after the decimal sign. One structure is selected at random from each bin and placed in the reduced ensemble $\Omega_{\mathrm{red}}$. This process is repeated for each identified cluster.

We note that the selector can control the size of the reduced ensemble by tuning the width of a bin/level. This approach indirectly biases the reduced ensemble by cluster size. Larger clusters with more structures result in more energy levels; therefore, more representative structures are selected from larger clusters to populate the reduced ensemble. Structure diversity retention is another indirect property of this approach as demonstrated experimentally in Section 3.2.

In Section 3, the $\Omega_{\mathrm{red}}$ ensemble selected as described above is compared against the reduced ensemble identified using truncation selection which does not employ clustering. To populate the reduced ensemble from the truncation-based approach, given a target size *M*, higher-energy structures are discarded to keep the *M* lowest-energy structures in an ensemble.

### 2.5. Datasets for Evaluation

We consider two datasets. The first is a benchmark dataset of 10 proteins of varying lengths (ranging from 53 to 123 amino acids) and folds (α, β, and α+β) that are widely used for evaluation by structure generation algorithms [12,16,17,52]. The second dataset contains 10 hard, free-modeling target domains from CASP12 and CASP13. To account for stochasticity, the HEA structure generation algorithm is run 5 times for each protein target; the structures generated in each run are aggregated to populate the $\Omega_{\mathrm{gen}}$ ensemble of 250,000 structures per target. In the clustering algorithms employed here to cluster the featurized $\Omega_{\mathrm{gen}}$ ensemble, determining the number of clusters takes most of the time. Including the structure generation stage, runtime varies between 7–16 h for a single run on one Intel Xeon E5- 2670 CPU with 2.6 GHz base processing speed and 100 GB of RAM. We note that all our implementations and analyses are carried out in Python. The scikit library is utilized to obtain access to the k-means, GMM, and hierarchical clustering algorithms.

### 2.6. Experimental Setup

The $\Omega_{\mathrm{gen}}$ and $\Omega_{\mathrm{red}}$ ensembles are compared by size, quality, and diversity. First, the evaluation in Section 3 compares the sizes of $\Omega_{\mathrm{gen}}$ and $\Omega_{\mathrm{red}}$ on each target protein. The experiment considers each of the four clustering algorithms to determine which algorithm is more effective at size reduction.

To evaluate the proposed selector in our approach against truncation selection, an $\Omega_{\mathrm{red}}$ ensemble obtained by k-means, GMM, hierarchical clustering, or gmx-cluster-usr is compared to the $\Omega_{\mathrm{red}}$ ensemble obtained via truncation selection. We choose the maximum size over $\Omega_{\mathrm{red}}$ identified by k-means, GMM, hierarchical clustering, and gmx-cluster-usr to set the target size *M* for truncation selection. As the results in Section 3 demonstrate, k-means and GMM result in larger, reduced ensembles compared to those obtained with the hierarchical clustering or gmx-cluster-usr; therefore, the size of the reduced ensemble in the truncation based-approach matches the size of the reduced ensemble obtained via k-means or GMM.

Second, the $\Omega_{\mathrm{gen}}$ and $\Omega_{\mathrm{red}}$ ensembles on each target protein are compared in terms of the distances of the structures in them from a known native structure. As is common practice in *de-novo* PSP, we utilize the popular least root-mean-squared-deviation (lRMSD) to measure these distances [53]. We note that lRMSD stands for least RMSD, which refers to a two-step process, first removing the rigid-body translation and rotation in three-dimensional space (rigid-body motions) between two structures under comparison and then calculating the weighted/average Euclidean distances of corresponding atoms in the structures. We report the lRMSD measured over the main carbon atoms (CA atoms). The evaluation in Section 3 compares the minimum, average, and standard deviation of lRMSDs of structures from the known native structure in the $\Omega_{\mathrm{gen}}$ and $\Omega_{\mathrm{red}}$ ensembles on each target protein.

Third, the $\Omega_{\mathrm{gen}}$ and $\Omega_{\mathrm{red}}$ ensembles on each target protein are compared in terms of their energetic profiles. While the native structure is a reliable reference structure for an lRMSD-based comparison between two ensembles, the same is not true for energy. The native structure, as obtained from the PDB, has higher energy than many computed structures, as it has not been refined for a particular energy function. Refining it until a local optimum of the energy function under consideration has been obtained will change the native structure, often in significant ways. Therefore, the evaluation in Section 3 compares the energy distributions of the $\Omega_{\mathrm{gen}}$ and $\Omega_{\mathrm{red}}$ ensembles directly, and relates observations regarding the minimum and average energies of the ensembles, as well as the maximum energy difference between two structures in an ensemble.

Finally, the lRMSD and energy distributions are related jointly. Each structure is visualized based on its lRMSD from the known native structure serving as one coordinate and its Rosetta score4 energy serving as the other coordinate. The so-called energy landscapes associated with the $\Omega_{\mathrm{gen}}$ and $\Omega_{\mathrm{red}}$ ensembles of a target protein are visualized, and superimpositions are utilized to identify possible regions of the landscape that the proposed approach may have discarded.

## 3. Results

### 3.1. Comparing Ensemble Sizes Pre- and Post Reduction

In Table 1, $\Omega_{\mathrm{gen}}$ and $\Omega_{\mathrm{red}}$ are first compared in terms of size over the benchmark dataset. The reduction percentage $(1-\frac{|\Omega_{\mathrm{red}}|}{|\Omega_{\mathrm{gen}}|})\cdot 100$% is also reported for each target. The reductions obtained by k-means range from 54% to 71%. The GMM reductions vary from 53% to 71%. Gmx-cluster-usr and hierarchical clustering result in more dramatic reductions of more than 72% and 77% on all targets respectively, and over 80% on 5/10 and 9/10 of the targets, respectively. Similar results are obtained over the CASP dataset, as shown in Table 2. Reductions of 59% and higher are obtained via k-means. Reductions obtained via GMM are comparable to those obtained via k-means. Reductions of 72% and higher are achieved via gmx-cluster-usr. Reductions of around 80% and higher are obtained via hierarchical clustering.

### 3.2. Comparing Distributions of lRMSDs from the Native Structure Pre- and Post Reduction

Table 3 compares the minimum, average, and standard deviation of lRMSDs of structures in the $\Omega_{\mathrm{red}}$ and $\Omega_{\mathrm{gen}}$ ensembles to the known native structure on each target in the benchmark dataset. The top panel of the table compares the minimum lRMSDs of the ensembles including the ensemble generated by truncation-based selection; the middle panel compares the average lRMSDs of the ensembles, and the bottom one compares the standard deviation of lRMSDs of structures in each ensemble to the known native structure per target protein. The minimum, average, and standard deviations over the generated ensembles are provided as reference in Column 2 (top, middle, and bottom panels, respectively). The difference of the (lRMSD) minimum, average, or standard deviation in $\Omega_{\mathrm{red}}$ over the corresponding quantity in $\Omega_{\mathrm{gen}}$ is reported in each setting.

Focusing on the *Diff.* columns listing differences in minimum lRMSDs, it is clear that truncation selection performs the worst in this regard; differences in minimum lRMSD range from 0.73 Å to 5.12 Å (see Column 12 of the top panel in Table 3). This means in the worst case, the best structure kept by truncation selection is 5.12 Å further away from the native structure than the best structure in the original ensemble. Truncation-based selection cannot maintain the quality of original ensemble.

In contrast, in the case of GMM and k-means, differences in minimum lRMSD (see Columns 4 and 6) are all 0 Å. The differences in minimum lRMSD for gmx-cluster-usr range from 0Å to 0.11 Å (see Column 10); for hierarchical clustering, the range is from 0 Å to 1.12 Å (see Column 8). This means that the structures closest to the native structure are always retained in the ensembles reduced by k-means and GMM. Not surprisingly, the slight increase in the differences when using gmx-cluster-usr and hierarchical clustering is the consequence of more drastic reduction in size of the reduced ensemble when using these two clustering algorithms over k-means or GMM.

The comparison shown in the middle panel of Table 3 indicates very little difference between the generated and reduced ensembles in terms of average lRMSDs. Column 4 shows the differences in average lRMSDs for k-means, which range from 0.41 Å to 0.60 Å. Column 6 shows an overall similar range for GMM (0.36 Å to 0.50 Å). Average lRMSD differences for hierarchical clustering range from 0.02 Å to 0.61 Å, as shown in Column 8. Column 10 shows that the differences in average lRMSD for gmx-cluster-usr range from 0.59 Å to 1.04 Å.

The comparison of differences on lRMSDs standard deviation for k-means is shown in Column 4 on the bottom panel and vary from 0.02 Å to 0.26 Å. These values are slightly different for GMM, ranging from 0.03 Å to 0.25 Å(see Column 6). As in the minimum lRMSD comparison, the differences obtained by gmx-cluster-usr and hierarchical clustering are slightly larger. Differences in standard deviation range from 0.23 Å to 0.53 Å for gmx-cluster-usr (shown in Column 10) and from 0 Å to 0.36 Å (with less than 0.1 Å on 7/10 targets; shown in Column 8) for hierarchical clustering.

Similar observations can be extracted from Table 4, which shows the performance over the CASP dataset. Table 4 confirms again that truncation selection loses the quality of the original ensemble in the reduced one. The quality of the reduced ensemble is preserved by all clustering algorithms, and the best results belong to k-means and GMM. All four clustering algorithms produce ensembles that have small differences in average lRMSDs and perform comparably in terms of standard deviation.

**Figure 4:**
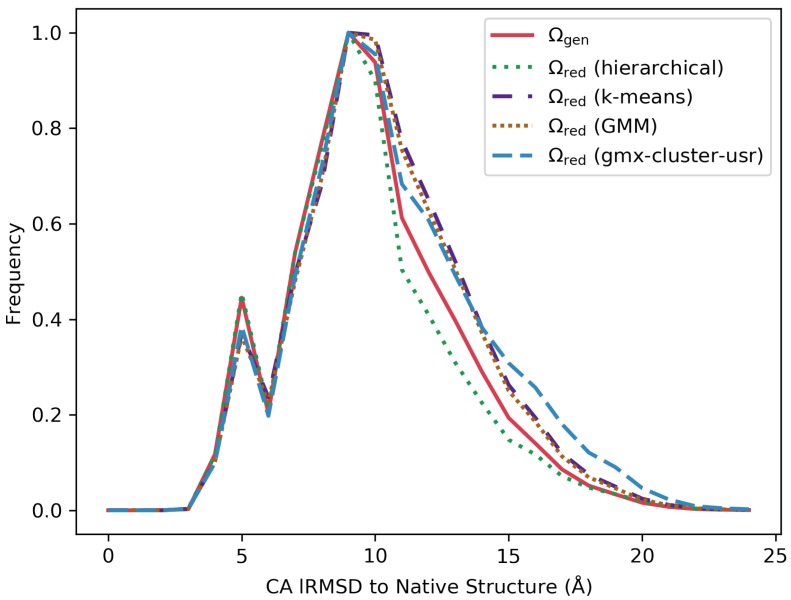
The distribution of structure lRMSDs from the native structure is shown for the $\Omega_{\mathrm{gen}}$ ensemble (in red) and the reduced $\Omega_{\mathrm{red}}$ ensembles obtained via k-means (purple), GMM (brown), hierarchical clustering (green), and gmx-cluster-usr (in blue). Results are shown for a representative target protein with native structure under PDB id 1ail.

Greater detail can be inferred from Figure 4, which shows results over a selected target protein (with native structure under PDB id 1ail). Figure 4 shows the actual distribution of structure lRMSDs from the native structure for the $\Omega_{\mathrm{gen}}$ ensemble along with the ensembles $\Omega_{\mathrm{red}}$ reduced via k-means, GMM, gmx-cluster-usr, and hierarchical clustering. Figure 4 shows that structures with similar relative frequencies of lRMSDs as in $\Omega_{\mathrm{gen}}$ are included in the reduced ensembles identified by each clustering algorithm.

### 3.3. Comparing Distributions of Energies Pre- and Post Reduction

Comparison of the minimum energy in an ensemble prior to and after reduction reveals some expected results. Since the proposed approach selects structures from each energy level, differences in the minimum energy in the $\Omega_{\mathrm{red}}$ and $\Omega_{\mathrm{gen}}$ ensembles of any target protein are negligible (less than 10^−2^). Since truncation selection retains the lowest-energy structure, as well, such differences are 0. Similar observations hold when comparing the energy diameters (the difference between the highest and lowest energy in an ensemble) of the $\Omega_{\mathrm{red}}$ and $\Omega_{\mathrm{gen}}$ ensembles on each target protein for the proposed approach. The differences are negligible (less than 10^−2^), as the proposed approach selects structures from all energy levels. The same is not true of truncation selection. The original ensemble contains many higher-energy structures, which truncation selection discards. As expected, for the same reasons, comparing the average energy yields small differences for the proposed approach while truncation selection results in large differences.

**Figure 5:**
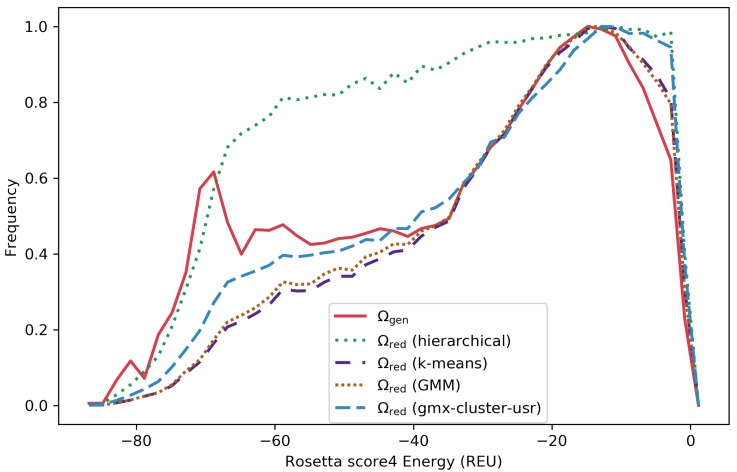
The distribution of Rosetta score4 energies is shown for the $\Omega_{\mathrm{gen}}$ ensemble (in red) and the reduced $\Omega_{\mathrm{red}}$ ensembles obtained via k-means (purple), GMM (brown), hierarchical (green), and gmx-cluster-usr clustering (in blue). Results are shown for the target protein with native structure under PDB id 1ail.

Figure 5 shows the distribution of structure energies for the $\Omega_{\mathrm{gen}}$ ensemble along with the $\Omega_{\mathrm{red}}$ ensembles reduced via k-means, GMM, gmx-cluster-usr, and hierarchical clustering for a selected target protein (with native structure under PDB id 1ail). Figure 5 shows that the relative frequencies of energies of the structures in the reduced ensembles are close to that of the structures in $\Omega_{\mathrm{gen}}$.

### 3.4. Visually Comparing Distributions of lRMSDs and Energies Pre- and Post Reduction

**Figure 6:**
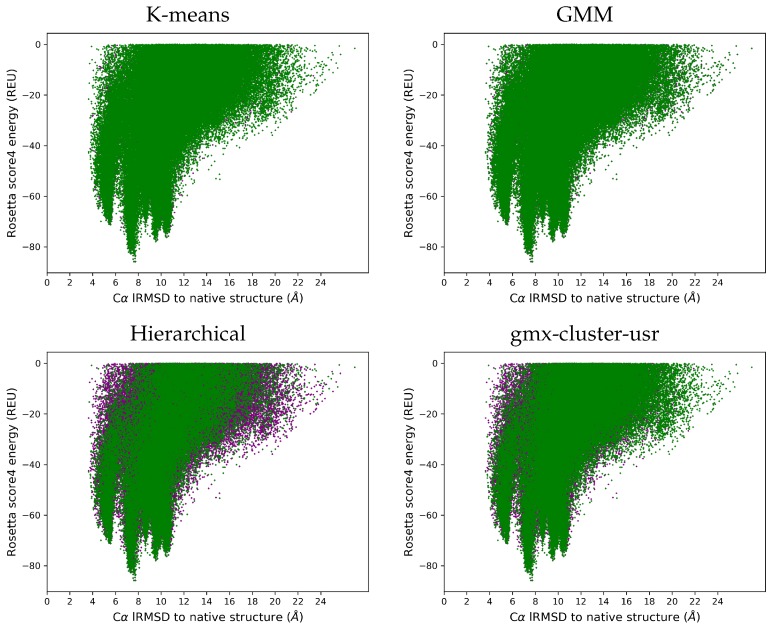
Benchmark Dataset: A representative target (with known native structure under PDB id 1ail) is selected. Structures in the $\Omega_{\mathrm{gen}}$ ensemble are plotted in purple in terms of their lRMSD (Å) from the native structure (X-axis) versus their Rosetta score4 energies (Y-axis) measured in Rosetta Energy Units (REUs). Structures in the $\Omega_{\mathrm{red}}$ ensemble are superimposed in green.

**Figure 7:**
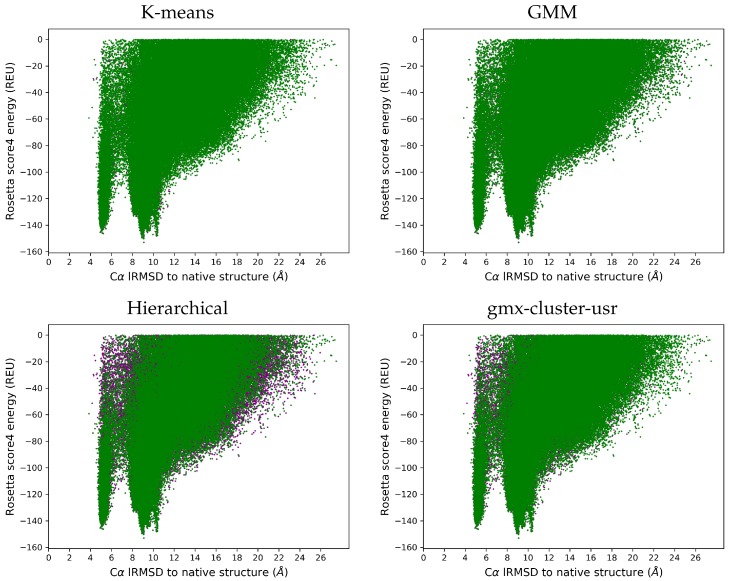
CASP Dataset: A representative protein (T1008-D1) is selected. Structures in the $\Omega_{\mathrm{gen}}$ ensemble are plotted in purple in terms of their lRMSD (Å) from the native structure (X-axis)versus their Rosetta score4 energies (Y-axis) measured in Rosetta Energy Units (REUs). Structures in the $\Omega_{\mathrm{red}}$ ensemble are superimposed in green.

We now compare the $\Omega_{\mathrm{gen}}$ and $\Omega_{\mathrm{red}}$ ensembles on each target protein in terms of Rosetta score4 energies and lRMSDs to the native structure. While the Supplementary Material shows these landscape figures for each target, here we show one representative landscape on each dataset (benchmark and CASP) that illustrates the behavior of each of the clsutering algorithms. Structures in $\Omega_{\mathrm{gen}}$ are drawn in purple, while those in the $\Omega_{\mathrm{red}}$ ensemble are drawn in green. Figure 6 does so for the benchmark dataset, and Figure 7 does so for the CASP dataset.

Figure 6 and Figure 7 (and those shown for each target protein in the Supplementary Material) show that the reduced ensemble $\Omega_{\mathrm{red}}$ includes structures from all the regions in the structure space populated by the original ensemble $\Omega_{\mathrm{gen}}$. All the purple dots being occluded by the superimposition in the k-means and GMM case visually makes the case that these two clustering algorithms perform better than gmx-cluster-usr and hierarchical clustering. This is not surprising, as k-means and GMM preserve more of the original ensemble.

## 4. Discussion

In summary, the presented results make the case that all four clustering algorithms are able to drastically decrease the structure ensemble size while preserving the quality and diversity of the original ensemble. GMM and k-means behave equally well in this regard, while gmx-cluster-usr and hierarchical clustering reduces the size of the ensembles more significantly and in response is also more prone to sacrificing quality.

Experience in molecular structure modeling informs that the choice of representation is often key to the success of a method. Here we provide further analysis into what the USR features are capturing. We do via a simple correlation analysis, where we compare the distribution of the lRMSDs versus USR scores of computed tertiary structures to the native structure. USR score is calculated as the Euclidean distance in the 12-dimensional USR feature space for two structures. The Supplementary Material (Table S5) lists the Pearson’s correlation coefficient between the two distributions for each protein target (in both the benchmark and CASP datasets).

**Figure 8:**
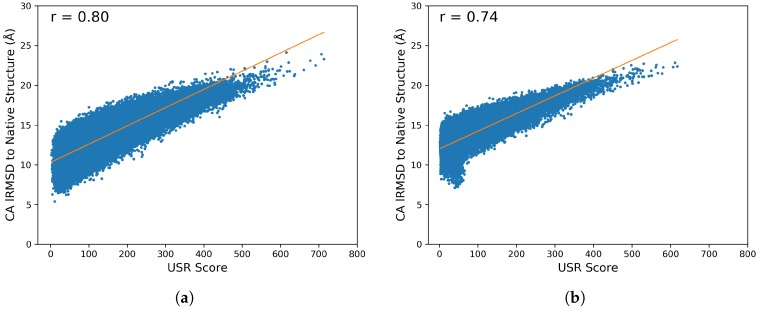
Correlation between USR scores and lRMSDs to the native structure of all tertiary structures computed on a target protein in the (**a**) benchmark dataset (with native structure under PDB id 1cc5) and (**b**) CASP dataset (with native structure under CASP id T0953s2-D3.)

Figure 8 plots the distributions against each-other for two targets that are representatives of the Pearson’s correlation coefficients obtained over targets in the benchmark and CASP datasets. Specifically, Figure 8a shows a correlation of 0.80 that is representative of what is observed over the benchmark dataset; Figure 8b shows a correlation of 0.74 that is representative of what is observed over the CASP dataset.

The median correlation over the benchmark dataset is 0.80, and the median correlation over the CASP dataset is 0.755. The correlations (representing what is observed over each of the datasets, with few outliers) show that the USR score is informative and a good proxy for lRMSD; we recall that the USR representation is also invariant to rigid-body motions, unlike Cartesian coordinate-based representations. Altogether, these results inform that the choice of the USR-based representation of tertiary structures is advantageous, allowing clustering algorithms to capture important structural differences that are then retained in the reduced ensemble by the selector.

## 5. Conclusions

The findings presented in this paper suggest that it is possible to significantly reduce the number of generated decoys without sacrificing quality and diversity. A three-stage approach relying on featurization, clustering, and selection is shown effective at doing so independent of the particular clustering algorithm employed. Various clustering algorithms are evaluated in the proposed approach.

The presented work opens up many venues of further research. An interesting application of this work can be in preparing data for model assessment methods, particularly in cases where datasets are expected to be highly imbalanced and so affect the performance of these methods. Other directions concern evaluating more clustering algorithms, including, for instance, clique finding algorithms. As often the case in molecular structure modeling and analysis literature, an important direction remains the choice of representation. Other future work can therefore investigate the utility and effectiveness of different features to represent structures, as well as consider advances in subspace clustering to address the high-dimensionality of molecular structure spaces.

## Figures and Tables

**Table 1:** $\Omega_{\mathrm{gen}}$ and $\Omega_{\mathrm{red}}$ are compared in terms of size over the benchmark dataset. The PDB ids, lengths, and  folds of the dataset are shown in Columns 1–3. Column 4 shows the size of $\Omega_{\mathrm{gen}}$. The size of $\Omega_{\mathrm{red}}$ and the reduction of $\Omega_{\mathrm{red}}$ over $\Omega_{\mathrm{gen}}$ are shown in Columns 5–12 for all clustering algorithms.

				K-Means		GMM		Hierarchical		Gmx-Cluster-Usr	
PDB Id	Length	Fold	$|\Omega_{\mathrm{gen}}|$	$|\Omega_{\mathrm{red}}|$	Red. (%)	$|\Omega_{\mathrm{red}}|$	Red. (%)	$|\Omega_{\mathrm{red}}|$	Red. (%)	$|\Omega_{\mathrm{red}}|$	Red. (%)
1ail	70	α	250 K	94,867	62.05	99,707	60.12	32,432	87.03	48,450	80.62
1bq9	53	β	250 K	79,181	68.33	77,873	68.85	24,705	90.12	39,716	84.11
1c8ca	64	β	250 K	87,209	65.12	88,437	64.63	29,795	88.08	46,817	81.27
1cc5	83	α	250 K	97,878	60.85	101,589	59.36	36,630	85.35	55,673	77.73
1dtja	76	α+β	250 K	75,421	69.83	79,134	68.35	29,506	88.2	41,456	83.42
1hhp	99	β	250 K	71,926	71.23	71,390	71.44	27,208	89.12	42,226	83.11
1tig	88	α+β	250 K	94,656	62.14	97,010	61.2	40,145	83.94	57,033	77.19
2ezk	93	α	250 K	114,244	54.3	115,929	53.63	49,509	80.2	62,439	75.02
2h5nd	123	α	250 K	110,196	55.92	111,353	55.46	55,153	77.94	67,671	72.93
3gwl	106	α	250 K	101,827	59.27	105,214	57.91	46,480	81.41	63,116	74.75

**Table 2:** $\Omega_{\mathrm{gen}}$ and $\Omega_{\mathrm{red}}$ are compared in terms of size over the CASP dataset. The CASP ids and lengths are shown in Columns 1–2. Column 3 shows the size of $\Omega_{\mathrm{gen}}$. The size of $\Omega_{\mathrm{red}}$ and the reduction of $\Omega_{\mathrm{red}}$ over $\Omega_{\mathrm{gen}}$ are shown in Columns 4–11 for all clustering algorithms.

			K-Means		GMM		Hierarchical		Gmx-Cluster-Usr	
CASP Id	Length	$|\Omega_{\mathrm{gen}}|$	$|\Omega_{\mathrm{red}}|$	Red. (%)	$|\Omega_{\mathrm{red}}|$	Red. (%)	$|\Omega_{\mathrm{red}}|$	Red. (%)	$|\Omega_{\mathrm{red}}|$	Red. (%)
T0859-D1	129	250 K	91,236	63.51	94,014	62.39	32,060	87.18	50,903	79.64
T0886-D1	69	250 K	72,351	71.06	88,986	64.41	27,328	89.07	42,397	83.04
T0892-D2	110	250 K	89,943	64.02	92,200	63.12	39,669	84.13	55,482	77.81
T0897-D1	138	250 K	98,262	60.7	101,119	59.55	50,352	79.86	68,703	72.52
T0898-D2	55	250 K	67,046	73.18	67,283	73.09	21,332	91.47	35,053	85.98
T0953s1-D1	67	250 K	51,078	79.57	50,509	79.8	16,417	93.43	29,690	88.12
T0953s2-D3	93	250 K	73,191	70.72	74,974	70.01	22,143	91.14	38,372	84.65
T0957s1-D1	108	250 K	92,028	63.19	93,872	62.45	38,665	84.53	54,951	78.02
T0960-D2	84	250 K	53,388	78.64	52,136	79.15	22,171	91.13	32,548	86.98
T1008-D1	77	250 K	101,433	59.43	105,360	57.86	51,809	79.28	68,428	72.63

**Table 3:** Comparison of minimum, average, and standard deviation of lRMSDs (to the known native structure) of structures in the $\Omega_{\mathrm{gen}}$ and $\Omega_{\mathrm{red}}$ ensembles of each target in the benchmark dataset. Comparison of minimum lRMSDs includes the ensemble reduced via truncation selection. Differences between the minimum, average, and standard deviation obtained over $\Omega_{\mathrm{red}}$ from those obtained over $\Omega_{\mathrm{gen}}$ are also related.

	**Minimum lRMSD (Å)**										
		**K-Means**		**GMM**		**Hierarchical**		**Gmx-Cluster-Usr**		**Truncation**	
**PDB Id**	$\Omega_{\mathrm{gen}}$	$\Omega_{\mathrm{red}}$	**Diff.**	$\Omega_{\mathrm{red}}$	**Diff.**	$\Omega_{\mathrm{red}}$	**Diff.**	$\Omega_{\mathrm{red}}$	**Diff.**	$\Omega_{\mathrm{red}}$	**Diff.**
1ail	3.64	3.64	0	3.64	0	3.64	0	3.64	0	4.37	0.73
1bq9	5.42	5.42	0	5.42	0	5.47	0.05	5.47	0.05	7.31	1.89
1c8ca	4.43	4.43	0	4.43	0	4.43	0	4.43	0	7.86	3.43
1cc5	5.4	5.4	0	5.4	0	6.52	1.12	5.4	0	7.85	2.45
1dtja	4.19	4.19	0	4.19	0	4.19	0	4.19	0	9.31	5.12
1hhp	11	11	0	11	0	11.29	0.29	11.02	0.02	12.88	1.88
1tig	5.34	5.34	0	5.34	0	5.45	0.11	5.45	0.11	6.59	1.25
2ezk	3.41	3.41	0	3.41	0	3.41	0	3.41	0	5.09	1.68
2h5nd	10.32	10.32	0	10.32	0	10.32	0	10.32	0	11.9	1.58
3gwl	4.85	4.85	0	4.85	0	4.85	0	4.85	0	7.81	2.96
	**Average lRMSD (Å)**										
		**K-Means**		**GMM**		**Hierarchical**		**Gmx-Cluster-Usr**			
**PDB Id**	$\Omega_{\mathrm{gen}}$	$\Omega_{\mathrm{red}}$	**Diff.**	$\Omega_{\mathrm{red}}$	**Diff.**	$\Omega_{\mathrm{red}}$	**Diff.**	$\Omega_{\mathrm{red}}$	**Diff.**		
1ail	10.42	10.89	0.47	10.85	0.43	10.19	0.23	11.17	0.75		
1bq9	9.76	10.18	0.42	10.17	0.41	9.74	0.02	10.39	0.63		
1c8ca	12.29	12.75	0.46	12.72	0.43	12.25	0.04	12.96	0.67		
1cc5	12.53	12.94	0.41	12.89	0.36	12.5	0.03	13.12	0.59		
1dtja	11.87	12.35	0.48	12.29	0.42	11.95	0.08	12.5	0.63		
1hhp	15.56	16.06	0.5	16.03	0.47	15.85	0.29	16.31	0.75		
1tig	12.85	13.36	0.51	13.31	0.46	12.81	0.04	13.81	0.96		
2ezk	10.17	10.77	0.6	10.72	0.55	10.78	0.61	11.21	1.04		
2h5nd	15.79	16.24	0.45	16.2	0.41	16.16	0.37	16.51	0.72		
3gwl	12.44	13.02	0.58	12.94	0.5	12.68	0.24	13.34	0.9		
	**Standard Deviation lRMSD (Å)**											
		**K-Means**		**GMM**		**Hierarchical**		**Gmx-Cluster-Usr**			
**PDB Id**	$\Omega_{\mathrm{gen}}$	$\Omega_{\mathrm{red}}$	**Diff.**	$\Omega_{\mathrm{red}}$	**Diff.**	$\Omega_{\mathrm{red}}$	**Diff.**	$\Omega_{\mathrm{red}}$	**Diff.**		
1ail	3.11	3.2	0.09	3.17	0.06	3.11	0	3.49	0.38		
1bq9	1.78	1.76	0.02	1.89	0.11	1.89	0.11	2.11	0.33		
1c8ca	2.18	2.23	0.05	2.22	0.04	2.15	0.03	2.45	0.27		
1cc5	2.31	2.34	0.03	2.34	0.03	2.35	0.04	2.54	0.23		
1dtja	2.08	2.31	0.23	2.27	0.19	2.15	0.07	2.44	0.36		
1hhp	1.82	1.94	0.12	1.93	0.11	1.9	0.08	2.08	0.26		
1tig	3.22	3.34	0.12	3.34	0.12	3.21	0.01	3.72	0.5		
2ezk	2.41	2.67	0.26	2.66	0.25	2.77	0.36	2.94	0.53		
2h5nd	2.03	2.22	0.19	2.21	0.18	2.3	0.27	2.46	0.43		
3gwl	2.9	3.02	0.12	3.01	0.11	2.99	0.09	3.22	0.32		

**Table 4:** Comparison of minimum, average, and standard deviation of distribution of lRMSDs (to the known native structure) of structures in the $\Omega_{\mathrm{gen}}$ and $\Omega_{\mathrm{red}}$ ensembles of each target in the CASP dataset. Comparison of minimum lRMSDs includes the ensemble reduced via truncation selection. Differences between the minimum, average, and standard deviation obtained over $\Omega_{\mathrm{red}}$ from those obtained over $\Omega_{\mathrm{gen}}$ are also related.

	**Minimum lRMSD (Å)**										
		**K-Means**		**GMM**		**Hierarchical**		**Gmx-Cluster-Usr**		**Truncation**	
**CASP Id**	$\Omega_{\mathrm{gen}}$	$\Omega_{\mathrm{red}}$	**Diff.**	$\Omega_{\mathrm{red}}$	**Diff.**	$\Omega_{\mathrm{red}}$	**Diff.**	$\Omega_{\mathrm{red}}$	**Diff.**	$\Omega_{\mathrm{red}}$	**Diff.**
T0859-D1	11.37	11.37	0	11.37	0	11.96	0.59	11.96	0.59	13.12	1.75
T0886-D1	7.96	7.96	0	7.96	0	8.73	0.77	8.73	0.77	11.24	3.28
T0892-D2	7.71	7.71	0	7.71	0	8.28	0.57	7.71	0	9.11	1.4
T0897-D1	10.18	10.18	0	10.18	0	10.64	0.46	10.64	0.46	11.62	1.44
T0898-D2	7.51	7.51	0	7.51	0	7.51	0	7.51	0	8.68	1.17
T0953s1-D1	6.14	6.14	0	6.14	0	6.29	0.15	6.29	0.15	8.18	2.04
T0953s2-D3	7.13	7.13	0	7.13	0	7.24	0.11	7.24	0.11	8.17	1.04
T0957s1-D1	7.65	7.65	0	7.65	0	7.76	0.11	7.76	0.11	9.39	1.74
T0960-D2	7.26	7.26	0	7.26	0	7.26	0	7.26	0	8.12	0.86
T1008-D1	3.85	3.85	0	3.85	0	3.85	0	3.85	0	5.67	1.82
**Minimum lRMSD (Å)**											
		**K-Means**		**GMM**		**Hierarchical**		**Gmx-Cluster-Usr**			
**CASP Id**	$\Omega_{\mathrm{gen}}$	$\Omega_{\mathrm{red}}$	**Diff.**	$\Omega_{\mathrm{red}}$	**Diff.**	$\Omega_{\mathrm{red}}$	**Diff.**	$\Omega_{\mathrm{red}}$	**Diff.**		
T0859-D1	17.47	17.64	0.17	17.63	0.16	17.49	0.02	17.78	0.31		
T0886-D1	13.16	13.66	0.5	13.67	0.51	13.31	0.15	13.82	0.66		
T0892-D2	14.81	15.49	0.68	15.43	0.62	15.02	0.21	15.71	0.9		
T0897-D1	17.3	17.84	0.54	17.81	0.51	17.54	0.24	18.04	0.74		
T0898-D2	11.56	11.72	0.16	11.71	0.15	11.63	0.07	11.86	0.3		
T0953s1-D1	11.98	11.74	0.24	11.73	0.25	11.81	0.17	11.66	0.32		
T0953s2-D3	13.28	13.89	0.61	13.88	0.6	13.48	0.2	14.06	0.78		
T0957s1-D1	14.96	15.49	0.53	15.44	0.48	15.13	0.17	15.74	0.78		
T0960-D2	12.63	13.07	0.44	13.07	0.44	12.93	0.3	13.27	0.64		
T1008-D1	11.77	12.36	0.59	12.46	0.69	11.9	0.13	12.67	0.9		
**Minimum lRMSD (Å)**											
		**K-Means**		**GMM**		**Hierarchical**		**Gmx-Cluster-Usr**			
**CASP Id**	$\Omega_{\mathrm{gen}}$	$\Omega_{\mathrm{red}}$	**Diff.**	$\Omega_{\mathrm{red}}$	**Diff.**	$\Omega_{\mathrm{red}}$	**Diff.**	$\Omega_{\mathrm{red}}$	**Diff.**		
T0859-D1	1.83	1.84	0.01	1.9	0.07	1.91	0.08	1.99	0.16		
T0886-D1	1.67	1.82	0.15	1.79	0.12	1.69	0.02	1.98	0.31		
T0892-D2	3.02	2.98	0.04	2.97	0.05	3.04	0.02	3.25	0.23		
T0897-D1	2.75	2.74	0.01	2.73	0.02	2.76	0.01	2.92	0.17		
T0898-D2	1.03	1.17	0.14	1.16	0.13	1.14	0.11	1.26	0.23		
T0953s1-D1	1.51	1.51	0	1.51	0	1.5	0.01	1.49	0.02		
T0953s2-D3	1.9	1.84	0.06	1.83	0.07	1.86	0.04	2.06	0.16		
T0957s1-D1	3.04	3.04	0	3.03	0.01	3.04	0	3.23	0.19		
T0960-D2	1.85	1.94	0.09	1.95	0.1	1.92	0.07	2.05	0.2		
T1008-D1	3.7	3.72	0.02	3.69	0.01	3.74	0.04	3.94	0.24		

## Supplementary Materials

Supplementary Materials are available online.

## Abbreviations

The following abbreviations are used in this manuscript:

BIC	Bayesian Information Criterion
CASP	Critical Assessment of protein Structure Prediction
DB	Davies-Bouldin
GMM	Gaussian Mixture Model
HEA	Hybrid Evolutionary Algorithm
lRMSD	least Root-Mean-Squared-Deviation
PDB	Protein Data Bank
PSP	Protein Structure Prediction
SSE	Sum of Squared Errors
USR	Ultrafast Shape Recognition
GROMACS	GROningen MAchine for Chemical Simulations
