# Supplementary material for: Reducing Ensembles of Protein Tertiary Structures Generated De Novo via Clustering
Source: Molecules. 2020 May 9;25(9):2228. doi: 10.3390/molecules25092228 (PMC7248879; doi:10.3390/molecules25092228)
Supplement: Supplementary file 1 [file molecules-25-02228-s001.pdf]

Article

Supplementary Material

Reducing Ensembles of Protein Tertiary Structures Generated De Novo via Clustering

Ahmed Bin Zaman[1,†], Parastoo Kamranfar[1,†] Carlotta Domeniconi[1,†,*] and Amarda Shehu[1,2,3,4,†,*]

[1] Department of Computer Science, George Mason University, Fairfax, VA 22030, USA; azaman6@gmu.edu; pkamranf@gmu.edu; cdomenic@gmu.edu; amarda@gmu.edu
[2] Center for Advancing Human-Machine Partnerships, George Mason University
[3] Department of Bioengineering, George Mason University
[4] School of Systems Biology, George Mason University
* Correspondence: cdomenic@gmu.edu; amarda@gmu.edu
† Current address: 4400 University Dr., MS 4A5, Fairfax, VA 22030, USA.

Version May 1, 2020 submitted to Journal Not Specified

1. Comparing Distributions of lRMSDs from Known Native Structure over CASP Dataset

Figures 1, 2, and 3 respectively show the minimum, average, and standard deviation of lRMSDs (to the known native structure) of the structures in the original and the reduced ensembles for each target in the CASP dataset. Figure 1 includes the ensemble reduced via truncation selection as a baseline.

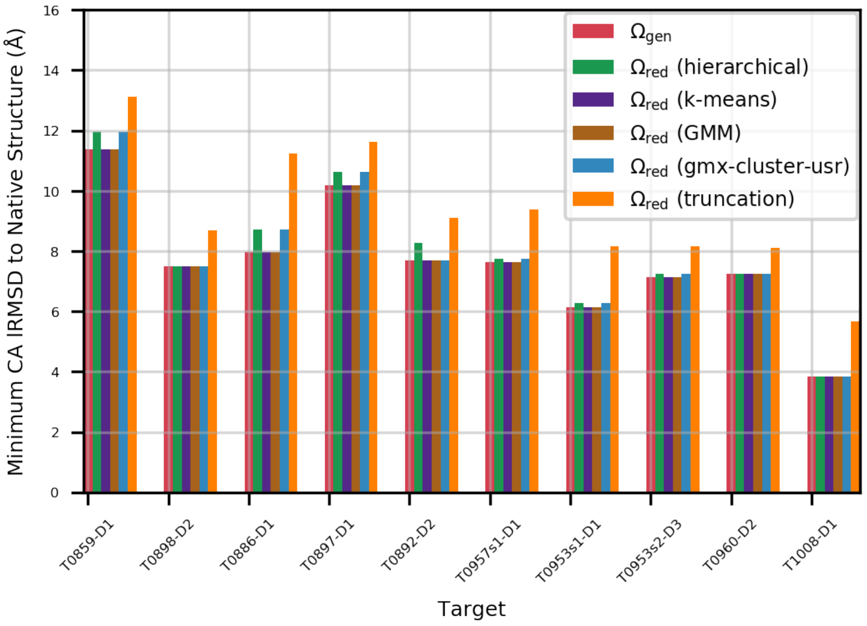

Figure 1. Comparison of minimum lRMSDs (to the known native structure) of structures in the Ω_{gen} and Ω_{red} ensembles of each target in the CASP dataset.

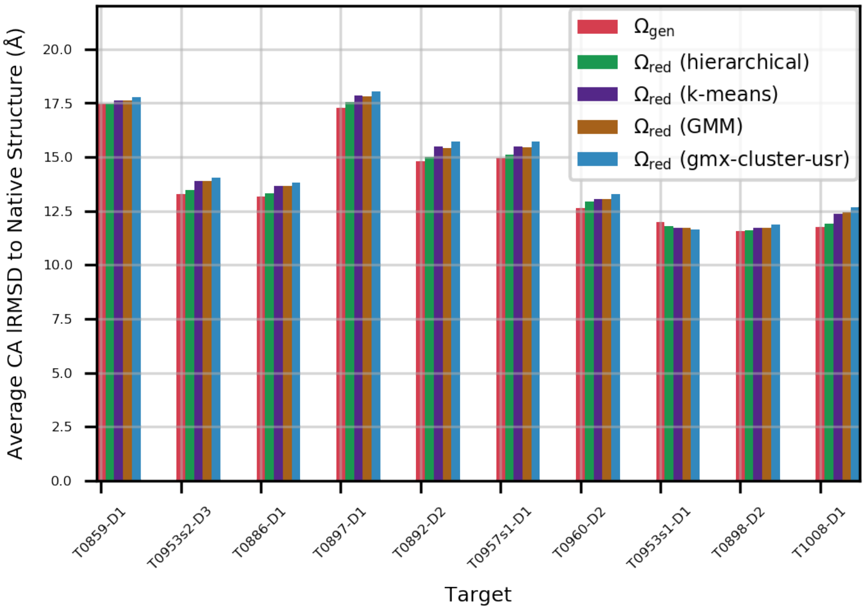

Figure 2. Comparison of average lRMSDs (to the known native structure) of structures in the Ω_{gen} and Ω_{red} ensembles of each target in the CASP dataset.

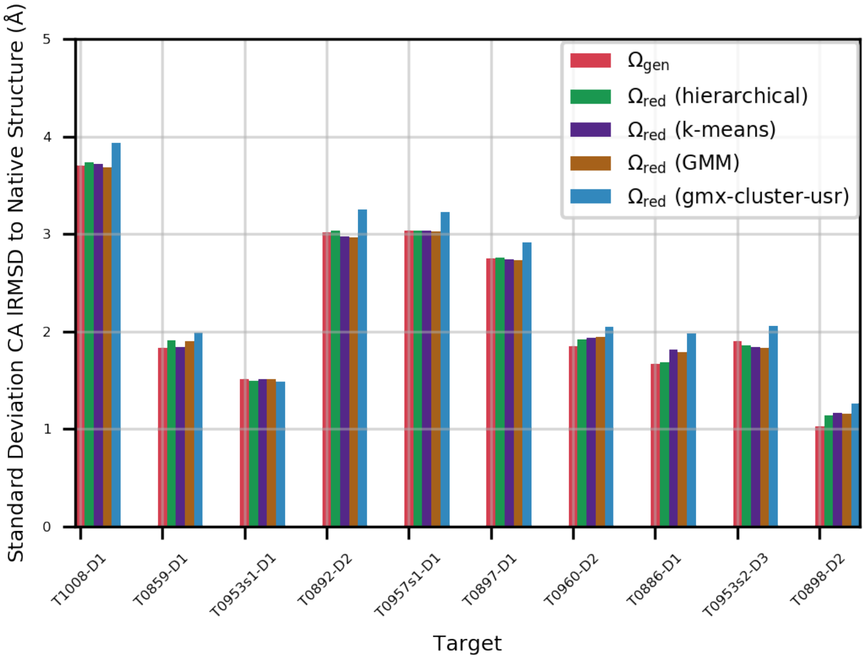

Figure 3. Comparison of standard deviation of distribution of lRMSDs (to the known native structure) of structures in the Ω_{gen} and Ω_{red} ensembles of each target in the CASP dataset.

1.1. Visually Comparing Distributions of lRMSDs and Energies Pre- and Post Reduction

The Ω_{gen} and Ω_{red} ensembles for the benchmark dataset are visualized in Figure 4-7. Rosetta score4 energy function values are plotted against the lRSMDs of the structures to the native structure. Structures in Ω_{gen} are highlighted in purple, while the green ones belong to the Ω_{red} ensembles. Figure 4 superimposes the Ω_{red} ensemble identified by k-means over the generated ensemble, and GMM-corresponding results are given in Figure 5. The Ω_{red} ensemble obtained via hierarchical clustering is indicated in Figure 6 while the Ω_{red} ensemble identified by gmx-cluster-usr is shown in Figure 7.

Similar visualization is provided for the CASP dataset in Figure 8-11. Figures 4-11 show that the reduced ensemble Ω_{red} includes structures from all the regions in the structure space populated by the original ensemble Ω_{gen}. All the purple dots being occluded by the superimposition in the k-means and GMM case visually makes the case that these two clustering algorithms perform better than gmx-cluster-usr and hierarchical clustering. As stated earlier, this is not surprising, as k-means and GMM preserve more of the original ensemble.

2. Relating the Number of Clusters

Regardless of which process is used to identify an optimal value for the number of clusters, this number varies for each target protein. Figure 12 shows the distribution of this number for each protein. Specifically, the distribution shown in Figure 12(a) is obtained from the SSE-based approach for k-means clustering over all target proteins (over both datasets). Figure 12(b)-(c) do so for the BIC- and DB-based approaches, respectively. Figure 12(d) shows the distribution for gmx-cluster-usr clustering for radius = 0.1.

Figure 12 shows that, for most of the target proteins, the number of clusters is in the $10-40$ range. This suggests that a large number of similar structures are present in the generated structure ensemble; therefore, finding the underlying structure to reduce the generated structure ensemble while retaining the diversity and quality is a reasonable goal. Table 1, 2, 3, and 4 show the specific number of clusters for each run of k-means, GMM, hierarchical, and gmx-cluster-usr clustering algorithms respectively for each target.

Table 1. Number of clusters determined for each run of the k-means clustering algorithm on each target.

	No. of Clusters for K-means				
**Id**	**Run 1**	**Run 2**	**Run 3**	**Run 4**	**Run 5**
1ail	20	30	20	20	25
1bq9	25	30	25	30	25
1c8ca	25	30	30	25	25
1cc5	25	25	25	20	30
1dtja	25	25	30	25	30
1hhp	25	30	25	30	23
1tig	25	23	25	22	30
2ezk	25	30	30	30	30
2h5nd	23	25	22	25	25
3gwl	23	25	23	25	25
T0859-D1	23	25	20	20	25
T0886-D1	23	20	23	25	30
T0892-D2	20	20	20	23	20
T0897-D1	20	25	20	23	25
T0898-D2	20	20	30	30	30
T0953s1-D1	30	25	23	20	25
T0953s2-D3	20	20	20	23	20
T0957s1-D1	20	23	20	25	23
T0960-D2	20	23	25	23	30
T1008-D1	23	20	20	25	20

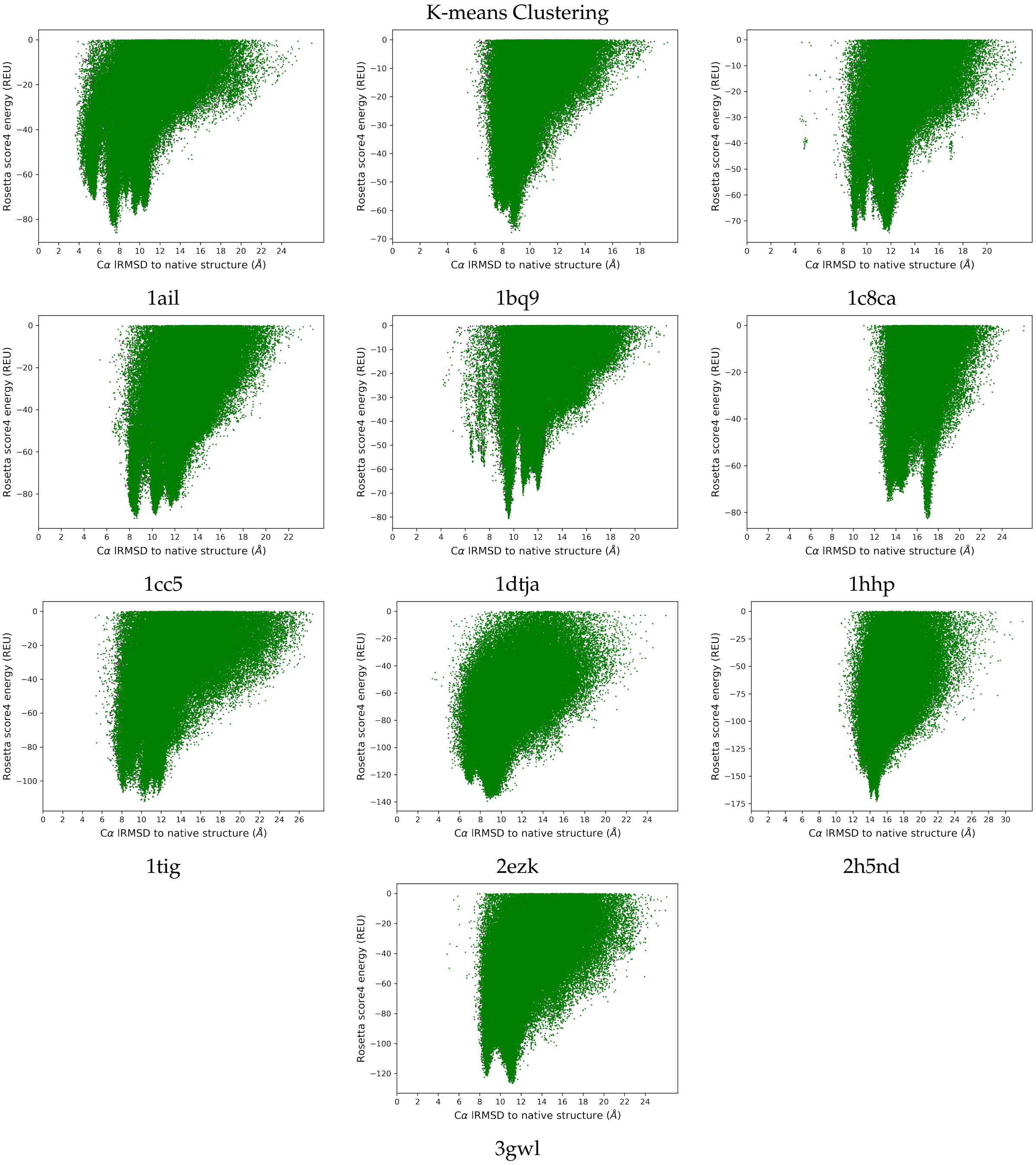

Figure 4. Benchmark Dataset: structures in the Ω_{gen} ensemble are plotted in purple in terms of their lRMSD (Å) from the native structure (x-axis) versus their Rosetta score4 energy function (y-axis) measured in Rosetta Energy Units (REUs). structures in the Ω_{red} ensemble obtained via k-means clustering are superimposed in green.

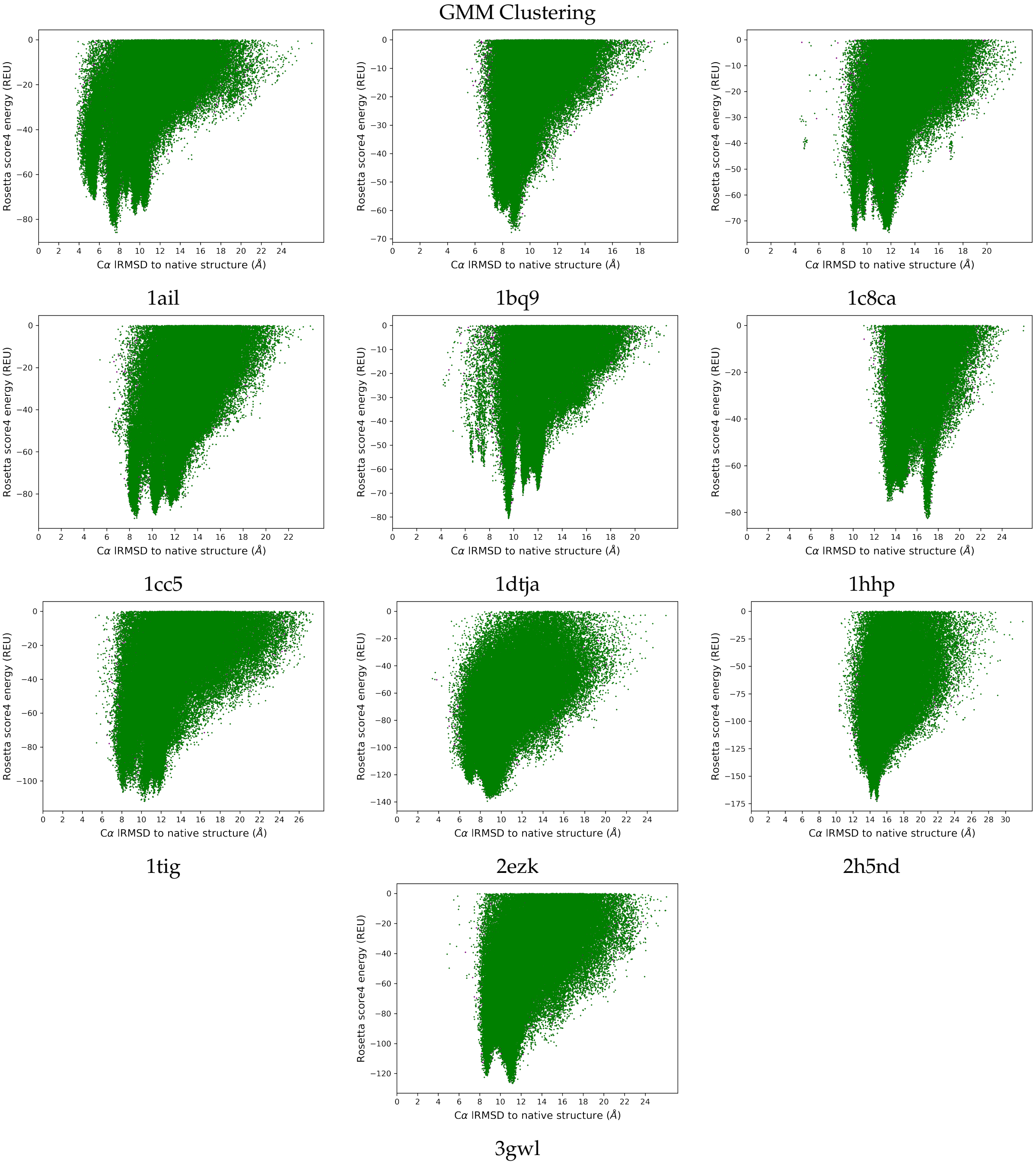

Figure 5. Benchmark Dataset: structures in the Ω_{gen} ensemble are plotted in purple in terms of their lRMSD (Å) from the native structure (x-axis) versus their Rosetta score4 energy function (y-axis) measured in Rosetta Energy Units (REUs). structures in the Ω_{red} ensemble obtained via GMM clustering are superimposed in green.

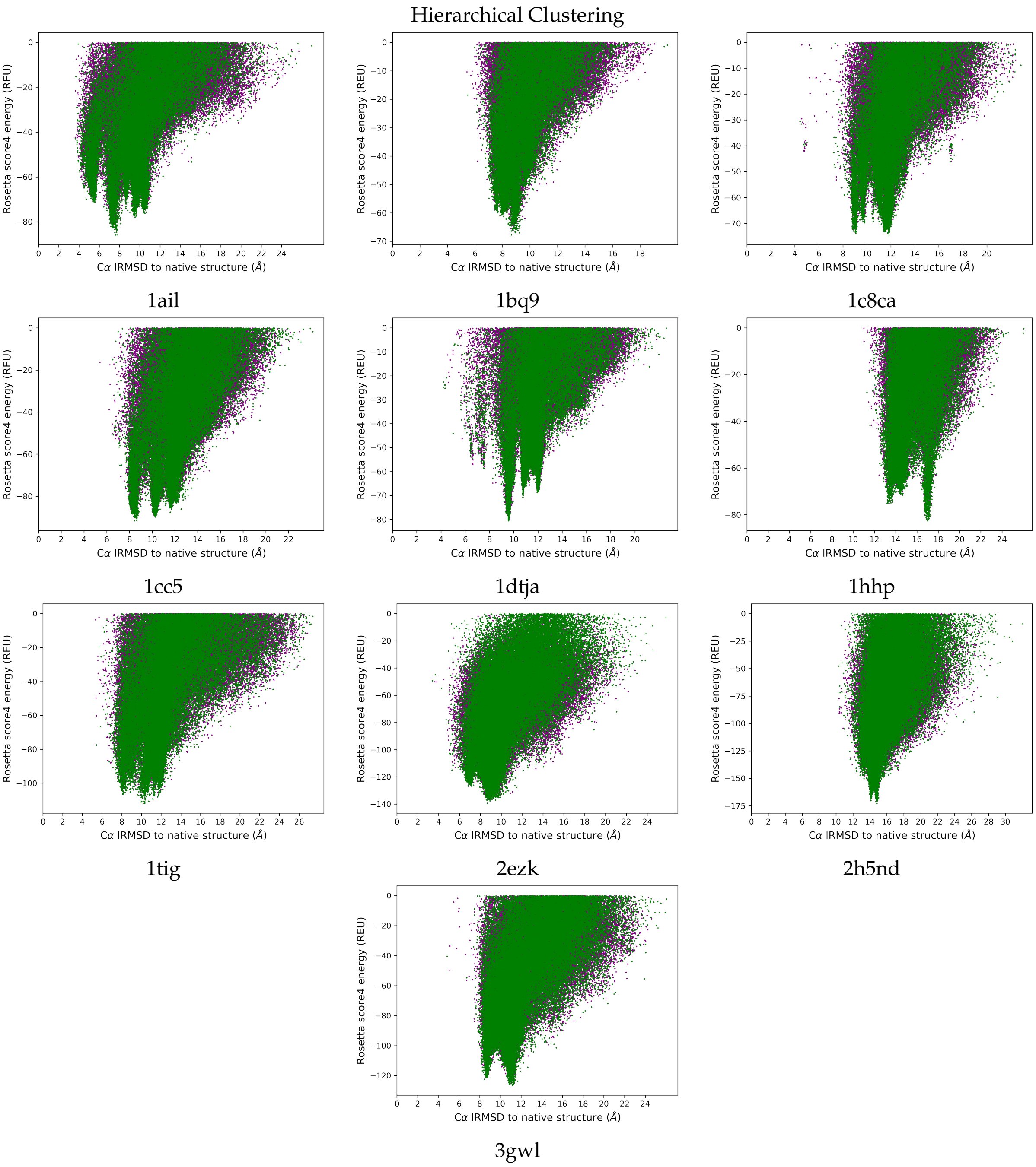

Figure 6. Benchmark Dataset: structures in the Ω_{gen} ensemble are plotted in purple in terms of their lRMSD (Å) from the native structure (x-axis) versus their Rosetta score4 energy function (y-axis) measured in Rosetta Energy Units (REUs). structures in the Ω_{red} ensemble obtained via hierarchical clustering are superimposed in green.

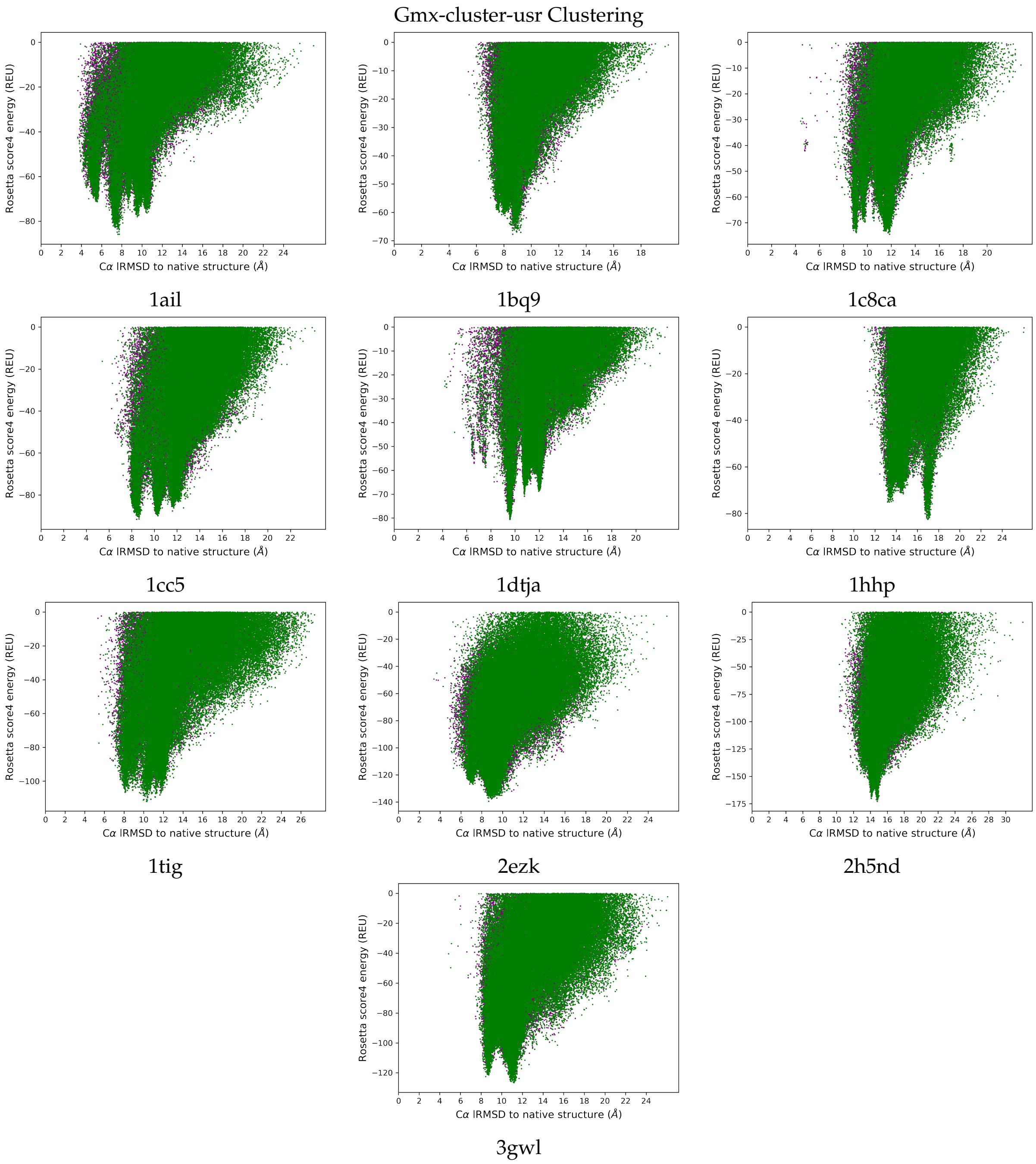

Figure 7. Benchmark Dataset: structures in the Ω_{gen} ensemble are plotted in purple in terms of their lRMSD (Å) from the native structure (x-axis) versus their Rosetta score4 energy function (y-axis) measured in Rosetta Energy Units (REUs). structures in the Ω_{red} ensemble obtained via gmx-cluster-usr are superimposed in green.

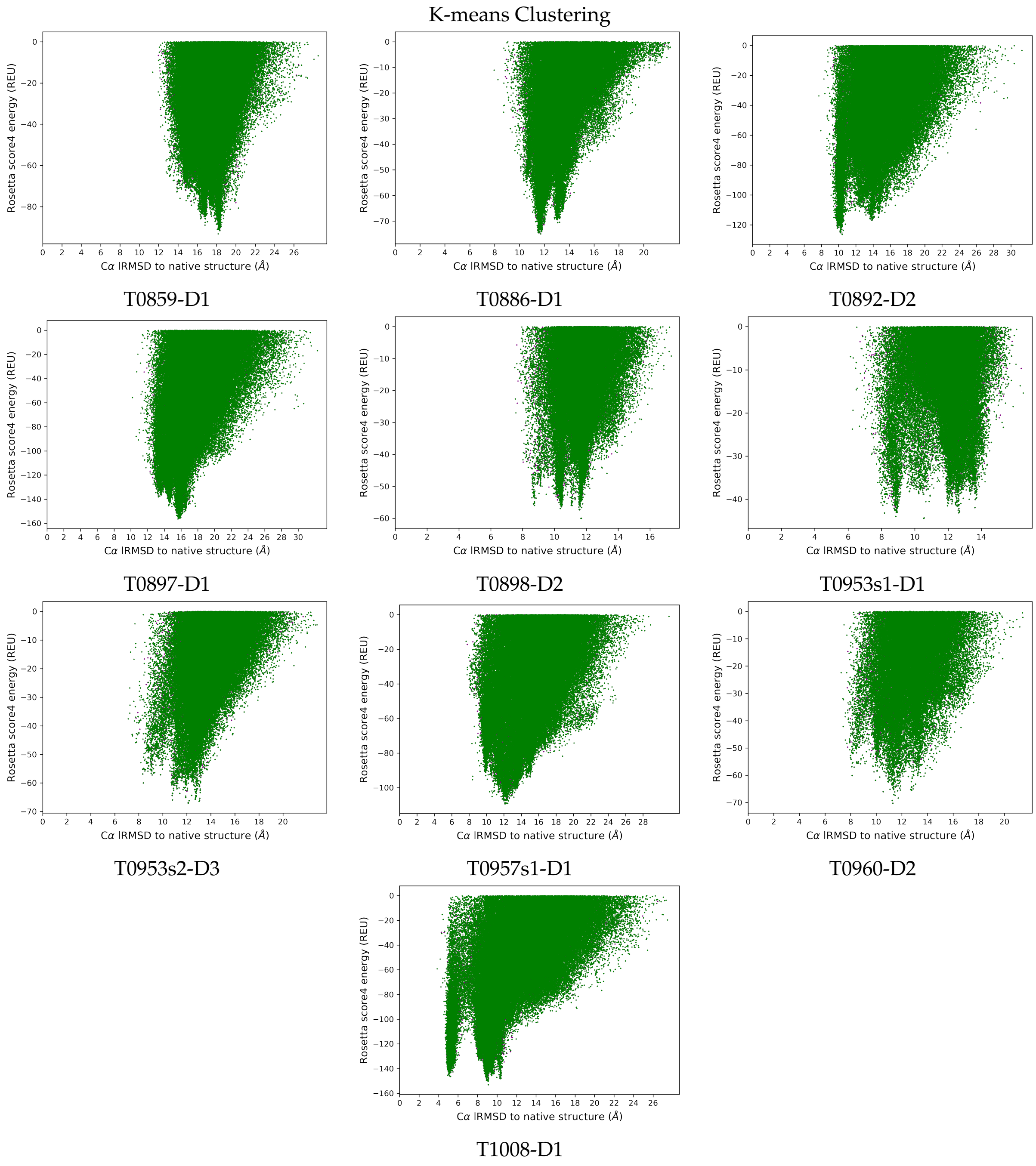

Figure 8. CASP Dataset: structures in the Ω_{gen} ensemble are plotted in purple in terms of their lRMSD (Å) from the native structure (x-axis) versus their Rosetta score4 energy function (y-axis) measured in Rosetta Energy Units (REUs). structures in the Ω_{red} ensemble obtained via k-means clustering are superimposed in green.

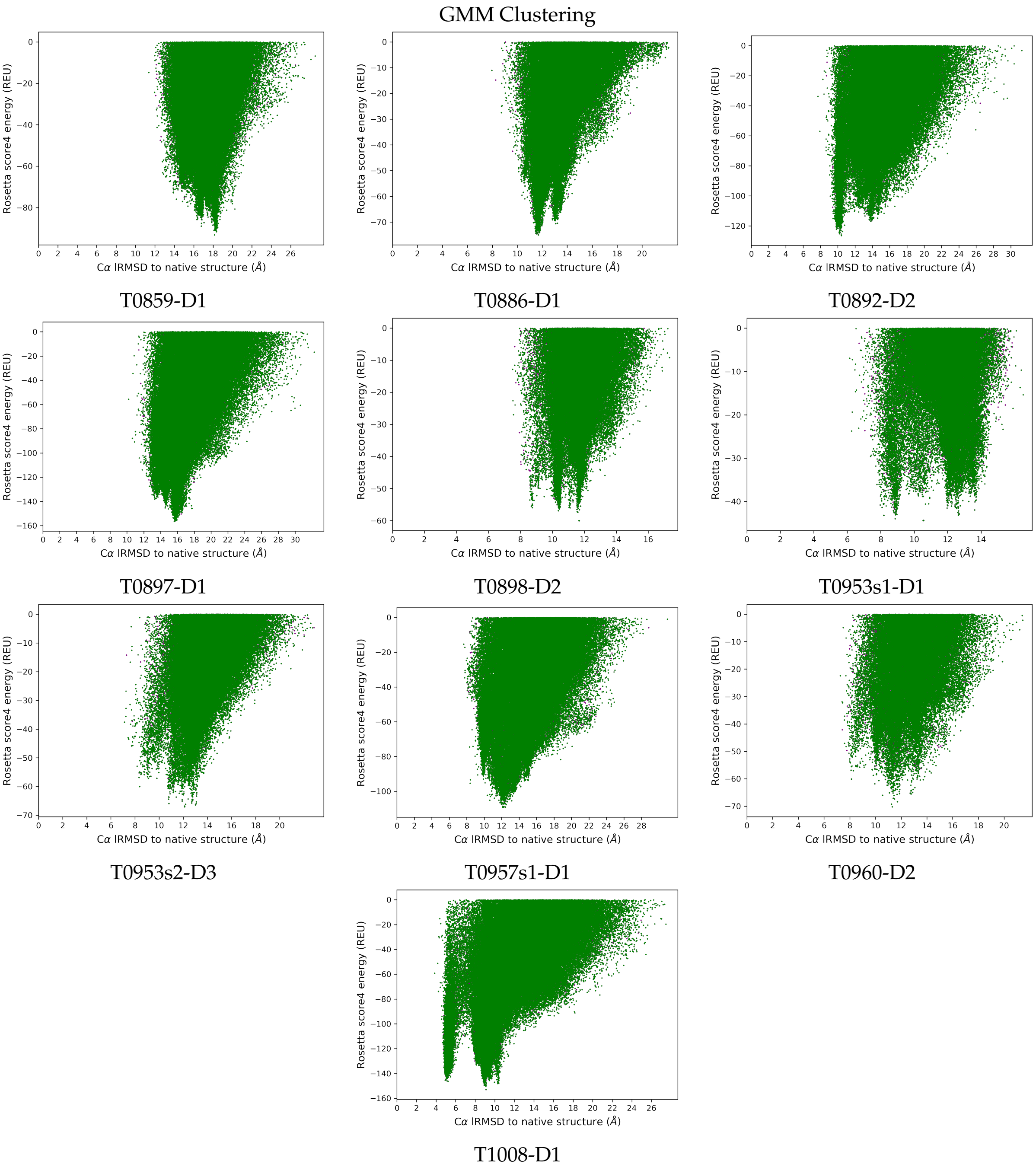

Figure 9. CASP dataset: structures in the Ω_{gen} ensemble are plotted in purple in terms of their lRMSD (Å) from the native structure (x-axis) versus their Rosetta score4 energy function (y-axis) measured in Rosetta Energy Units (REUs). structures in the Ω_{red} ensemble obtained via GMM clustering are superimposed in green.

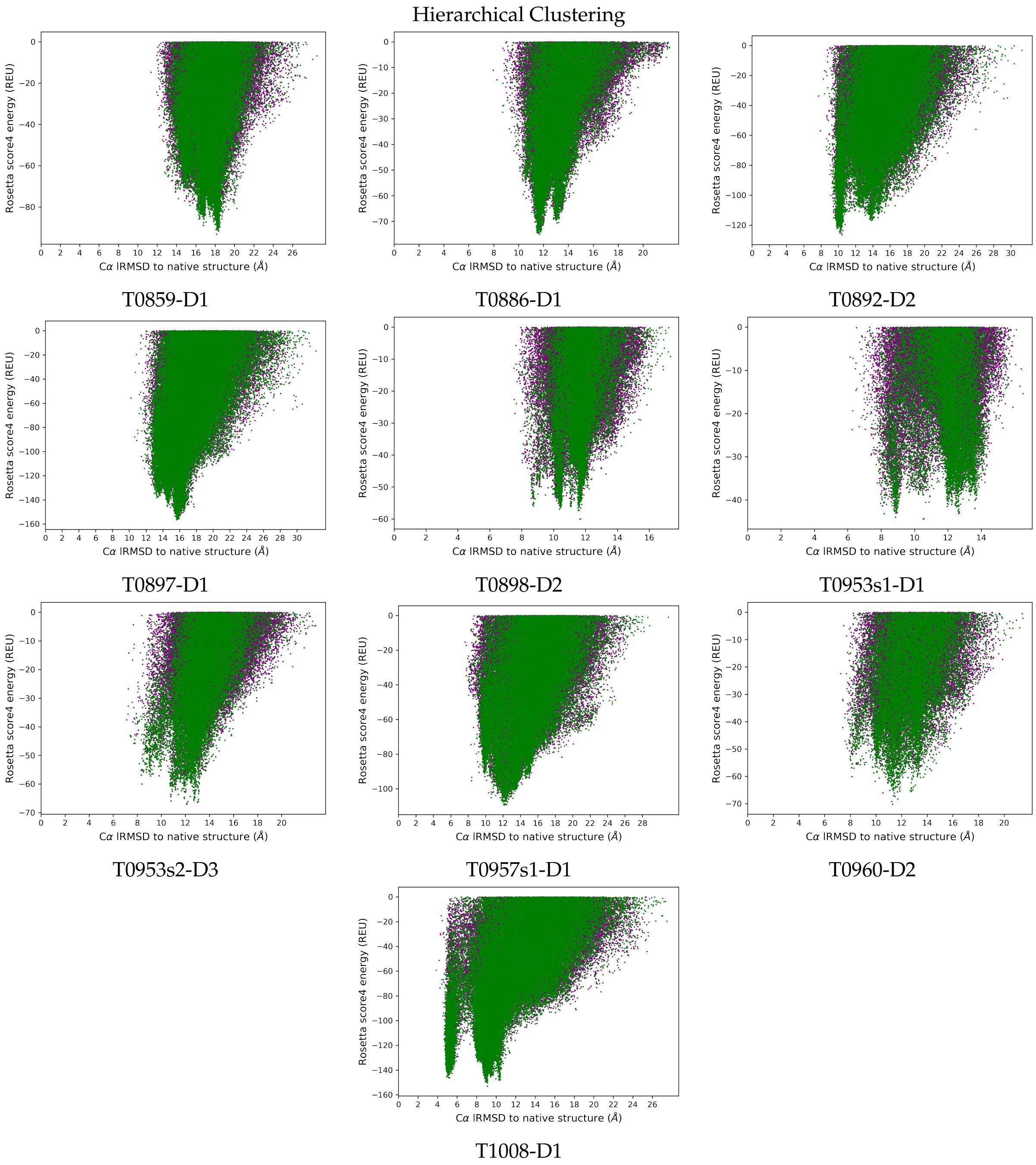

Figure 10. CASP dataset: structures in the Ω_{gen} ensemble are plotted in purple in terms of their lRMSD (Å) from the native structure (x-axis) versus their Rosetta score4 energy function (y-axis) measured in Rosetta Energy Units (REUs). structures in the Ω_{red} ensemble obtained via hierarchical clustering are superimposed in green.

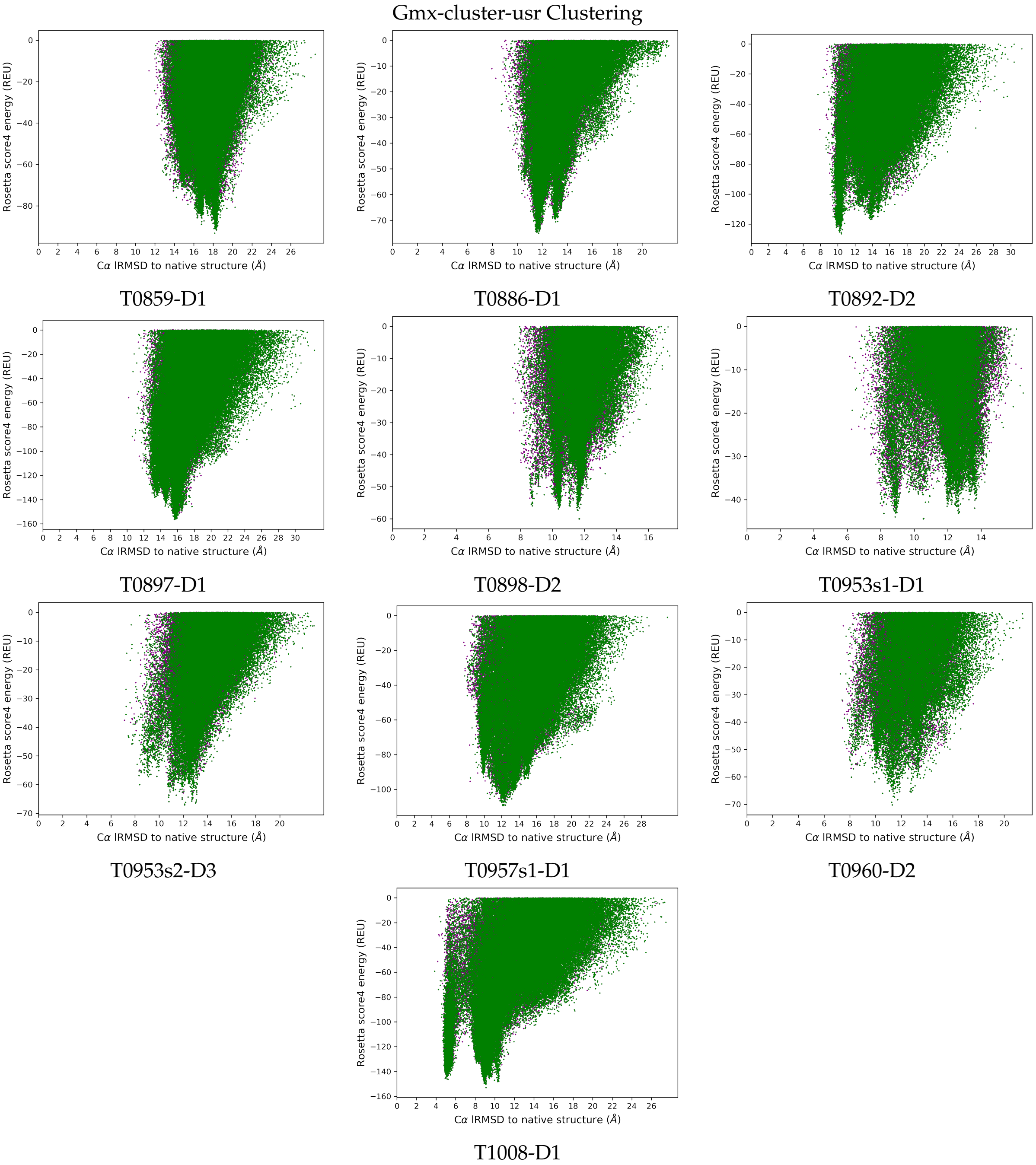

Figure 11. CASP dataset: structures in the Ω_{gen} ensemble are plotted in purple in terms of their lRMSD (Å) from the native structure (x-axis) versus their Rosetta score4 energy function (y-axis) measured in Rosetta Energy Units (REUs). structures in the Ω_{red} ensemble obtained via gmx-cluster-usr are superimposed in green.

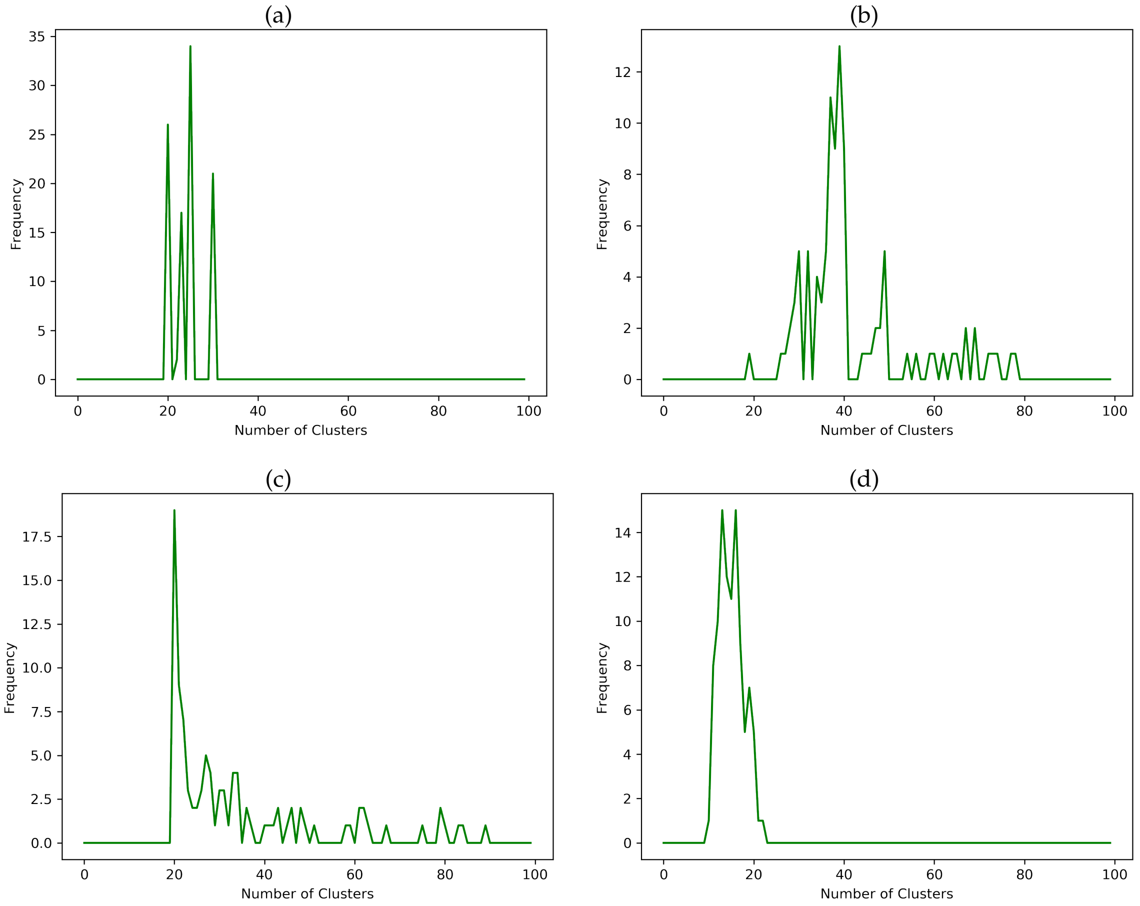

Figure 12. Distribution of the number of clusters identified via the (a) SSE-based process in k-means, (b) BIC-based process in GMM, (c) DB-based process in agglomerative hierarchical clustering, and (d) radius = 0.1 in gmx-cluster-usr clustering over target proteins in the benchmark and CASP datasets.

Table 2. Number of clusters determined for each run of the GMM clustering algorithm on each target.

	No. of Clusters for GMM				
**Id**	**Run 1**	**Run 2**	**Run 3**	**Run 4**	**Run 5**
1ail	44	47	46	49	45
1bq9	37	36	34	40	39
1c8ca	40	37	37	38	49
1cc5	78	39	39	40	67
1dtja	54	39	37	39	39
1hhp	48	27	40	49	32
1tig	30	59	39	40	69
2ezk	38	40	60	39	38
2h5nd	19	30	32	37	29
3gwl	30	29	56	49	74
T0859-D1	36	35	37	39	38
T0886-D1	38	29	39	37	32
T0892-D2	37	39	34	32	36
T0897-D1	47	40	36	65	67
T0898-D2	35	40	72	40	37
T0953s1-D1	30	34	64	39	38
T0953s2-D3	38	37	36	69	37
T0957s1-D1	26	35	37	28	77
T0960-D2	38	38	73	39	32
T1008-D1	62	48	30	28	34

3. Comparing lRSMD to USR Score

Table 5 shows the Pearson's correlation coefficient between USR scores and lRMSDs to the native structure of all the generated structures for each target in the benchmark and the CASP datasets.

© 2020 by the authors. Submitted to *Journal Not Specified* for possible open access publication under the terms and conditions of the Creative Commons Attribution (CC BY) license (http://creativecommons.org/licenses/by/4.0/).

Table 3. Number of clusters determined for each run of the hierarchical clustering algorithm on each target.

	No. of Clusters for Hierarchical				
**Id**	**Run 1**	**Run 2**	**Run 3**	**Run 4**	**Run 5**
1ail	20	21	67	34	23
1bq9	27	21	20	34	22
1c8ca	22	21	20	42	79
1cc5	84	28	21	20	89
1dtja	79	20	43	20	48
1hhp	40	22	34	45	23
1tig	26	51	24	27	62
2ezk	20	29	48	21	21
2h5nd	24	20	20	41	27
3gwl	25	22	62	36	75
T0859-D1	25	28	28	30	31
T0886-D1	22	33	26	20	20
T0892-D2	30	26	28	20	32
T0897-D1	46	46	31	49	61
T0898-D2	20	30	63	33	21
T0953s1-D1	20	23	61	37	36
T0953s2-D3	27	21	20	58	22
T0957s1-D1	20	27	33	31	80
T0960-D2	33	34	83	22	20
T1008-D1	59	43	21	20	20

Table 4. Number of clusters determined for each run of the gmx-cluster-usr clustering algorithm on each target.

	No. of Clusters for Gmx-cluster-usr				
**Id**	**Run 1**	**Run 2**	**Run 3**	**Run 4**	**Run 5**
1ail	15	15	17	11	13
1bq9	16	18	12	16	13
1c8ca	13	15	16	12	17
1cc5	19	13	16	14	17
1dtja	17	13	13	15	20
1hhp	19	14	20	16	13
1tig	16	15	18	13	19
2ezk	14	17	13	13	14
2h5nd	13	12	11	12	12
3gwl	12	19	13	17	15
T0859-D1	20	20	14	11	18
T0886-D1	15	13	17	20	18
T0892-D2	13	14	16	12	15
T0897-D1	16	16	18	16	19
T0898-D2	12	16	17	21	14
T0953s1-D1	17	19	15	16	22
T0953s2-D3	16	14	15	11	12
T0957s1-D1	14	14	10	14	14
T0960-D2	11	11	11	11	19
T1008-D1	16	12	13	15	16

Table 5. Pearson's correlation coefficient between USR scores and lRMSDs to the native structure of all generated structures for each target.

Id	Pearson's Coefficient
1ail	0.65
1bq9	0.71
1c8ca	0.80
1cc5	0.80
1dtja	0.70
1hhp	0.80
1tig	0.88
2ezk	0.71
2h5nd	0.85
3gwl	0.83
T0859-D1	0.49
T0886-D1	0.82
T0892-D2	0.85
T0897-D1	0.89
T0898-D2	0.59
T0953s1-D1	0.07
T0953s2-D3	0.74
T0957s1-D1	0.78
T0960-D2	0.59
T1008-D1	0.77
